# Supplementary material for: Environment-sensitive turn-on fluorescent probe enables live cell imaging of myeloperoxidase activity during NETosis
Source: Commun Chem. 2024 Nov 12;7:262. doi: 10.1038/s42004-024-01338-5 (PMC11557929; doi:10.1038/s42004-024-01338-5)
Supplement: Supplementary file 2 — Supplementary Information [file 42004_2024_1338_MOESM2_ESM.pdf]

Environment-Sensitive Turn-On Fluorescent Probe Enables Live Cell Imaging of Myeloperoxidase Activity during NETosis

Enebie Ramos Cáceres[1], Lotte Kemperman[1] and Kimberly M. Bonger[1]

[1]Department of Synthetic Organic Chemistry, Institute for Molecules and Materials, Radboud University, Heyendaalseweg 135, 6525AJ, Nijmegen, The Netherlands. E-mail: k.bonger@science.ru.nl

Table of contents

1. Supplementary Methods. 2

1.1 Materials and instruments. 2

1.2 General procedures for the synthesis of probes 1-6. 2

1.3 Measurement of ''turn-on'' of fluorescent probe 1 3

1.4 Probe 1 chemical stability. 3

1.5 Cell culture and differentiation. 3

1.6 NET quantification. 3

1.7 Oxidative burst. 3

1.8 Compound characterization data. 4

2. Supplementary Tables. 6

3. Supplementary Figures. 7

5. Supplementary References. 14

1. Supplementary Methods.

1.1 Materials and instruments.

Chemicals were purchased at Sigma Aldrich, TCI Europe, VWR chemicals, or Fisher Scientific and used without further purification. Water-sensitive reactions were performed in dry solvents in flame-dried glassware under positive flow of N_2. Solvents were dried by purging over activated alumina columns in an MBraun MB SPS800. Ultrapure Milli-Q water was obtained from QPOD Milli-Q system. Reactions were monitored using glass TLC plates (Merck, TLC Silica gel 60 F254) using UV absorption detection (254 nm) and by staining with potassium permanganate or ninhydrin solution and subsequent charring at 150 °C. Purification by flash column chromatography was executed using silica gel (VWR chemicals, 0.040-0.063 mm) or on a Biotage Selekt with 4-40 g silica gel cartridges (Screening Devices, 0.040-0.063 mm, 60 Å) or 25 g spherical C18 reverse-phase silica cartridges (Screening Devices, 0.020-0.045 mm, 100 Å). NMR spectra were recorded on a Bruker Avance III 400 MHz or 500 MHz and the compounds were assigned using ^{1}H NMR, ^{13}C{^{1}H} NMR (CPD or APT), 2D COSY, ^{1}H-^{13}C-HSQC, and ^{1}H-^{13}C-HMBC spectra. Chemical shifts were reported in parts per million (ppm) relative to the residual signal of the deuterated solvent. NMR data are presented in the following way: chemical shift (δ), multiplicity (s = singlet, bs = broad singlet, d = doublet, t = triplet, q = quartet, m = multiplet and/or multiple resonances), and coupling constants J in Hz. High resolution mass spectra (HRMS) were recorded on a Bruker timsTOF Pro 2. High performance liquid chromatography (HPLC) was performed on a Single-Quad Thermo ISQ equipped with a Thermo Scientific Accucore C18 (2.6 µm, 80 Å, 100x3 mm) column using 0.1% formic acid in acetonitrile (CH_3CN) and Milli-Q as eluents and UV absorbance (254 nm).

Fluorescence excitation and emission spectra were acquired on a Shimadzu RF-6000 spectrofluorometer. Extinction coefficients were calculated from absorbance measurements acquired on a Shimadzu UV-1900i UV-Vis spectrophotometer. All data was processed and analyzed using GraphPad Prism (v. 9.0.0).

Fluorescence measurements from assays were performed on a Tecan Spark M10 plate reader using Greiner Bio-One black PS F-bottom 96 well microplates. All data was processed and analyzed using GraphPad Prism (v. 9.0.0).

Microscopy images were captured using a Leica SP8x AOBS-WLL confocal microscope with a 63x/NA 1.4 objective using a 405 nm diode laser or 470-670 nm pulsed WLL and GaAsP PMT detector at a resolution of 2048x2048 or 4096x4096 pixels. Live cell imaging was performed in a controlled environment at 37 °C, 5% CO_2. Excitation wavelengths and emission windows were adjusted to the excitation/emission maxima of each probe. Probe **1** imaging was done using 405 nm as excitation wavelength and 480-560 nm as detection window. All images were processed with Fiji (ImageJ) and formatted with Adobe Illustrator. LUTs were chosen to represent and display the full range of data.

1.2 General procedures for the synthesis of probes 1-6.

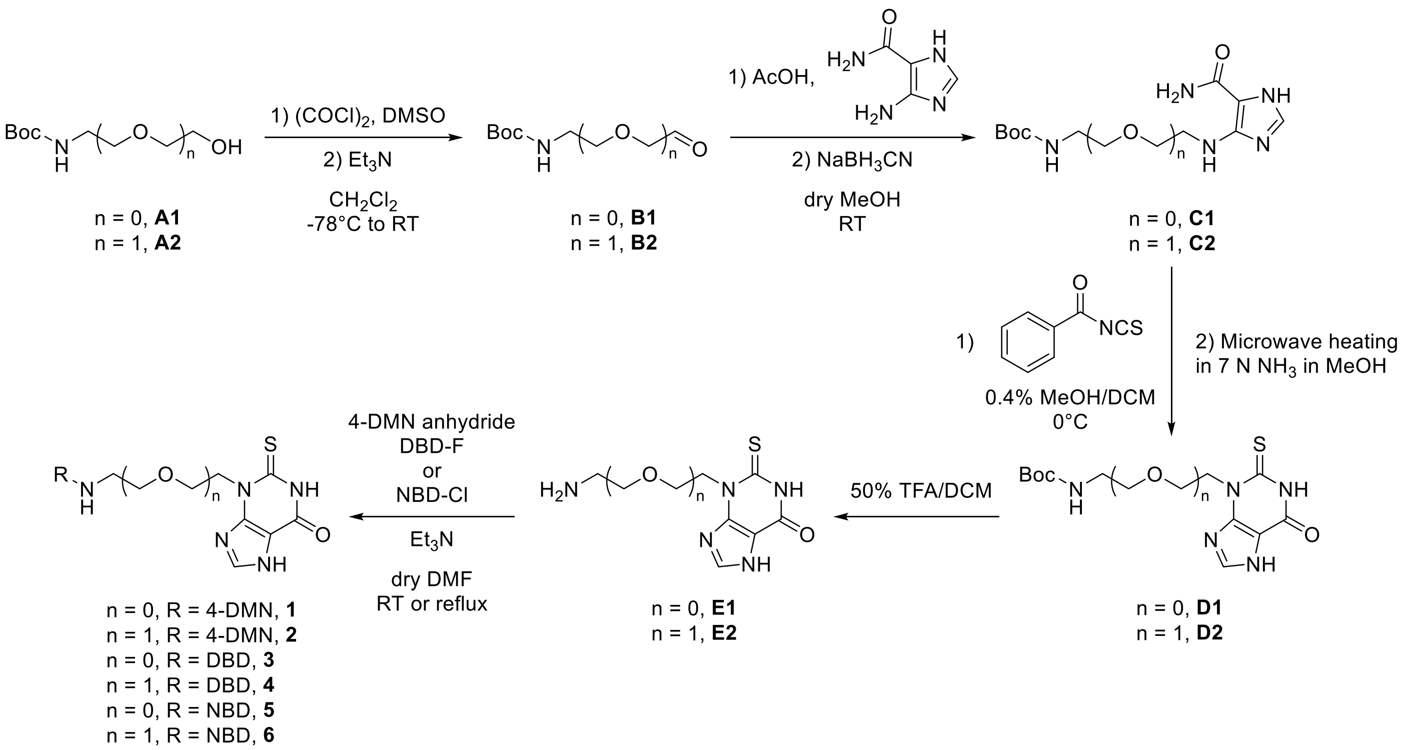

A solution of DMSO (14.8 mmol) in CH_2Cl_2 (5 mL) was added dropwise to a stirring solution of oxalyl chloride (7.4 mmol) in CH_2Cl_2 (10 mL) at -78 °C. After 10 min, *N*-Boc alcohol **A1** or **A2** (6.0 mmol) in CH_2Cl_2 (5 mL) was added dropwise and the resulting mixture was stirred for 30 min. Then, Et_3N (18.6 mmol) was added slowly and after 10 min the reaction was warmed up to room temperature and stirred for 2 h. Next, the mixture was quenched with sat. aq. NH_4Cl and extracted with CH_2Cl_2. The combined organic layers were dried over anhydrous $MgSO_4$, filtered, and concentrated *in vacuo* to give the corresponding *N*-Boc aldehyde **B1** or **B2**, which was used immediately in the next step without further purification.

To a stirring solution of the corresponding *N*-Boc aldehyde **B1** or **B2** (7.6 mmol) and AcOH (1.2 mmol) in dry CH_3OH (30 mL) was added 4-amino-1*H*-imidazole-5-carboxamide (6.0 mmol). The reaction was stirred for 2.5 h under N_2 before $NaBH_3CN$ (7.2 mmol) was added in one portion and the mixture was left stirring overnight under N_2. A solution of sat. aq. $NaHCO_3$ was added and most of the CH_3OH was removed *in vacuo*. The residue was extracted with EtOAc and the combined organic layers were dried over $MgSO_4$, filtered, and the crude product purified by flash silica gel column chromatography (0-10% CH_3OH/CH_2Cl_2) to give **C1** or **C2**.

To a stirring solution of **C1** or **C2** (2.4 mmol) in 0.4% CH_3OH/CH_2Cl_2 (3 mL) at 0 °C was added benzoyl isothiocyanate (2.6 mmol). After being stirred for 3 h, the volatiles were evaporated and the residue re-dissolved in 7 N NH_3 in CH_3OH. The solution was subjected to microwave heating at 80 °C for 5 h. After this time, the volatiles were evaporated *in vacuo* and the crude product purified by flash silica gel column chromatography (0-20% CH_3OH/CH_2Cl_2) to give **D1** or **D2**. Subsequently, **D1** or **D2** was treated with 50% TFA/CH_2Cl_2 (5 mL) and stirred for 2 h. The mixture was concentrated and cold Et_2O was added. The white precipitate was washed three times with cold Et_2O and dried *in vacuo* to give **E1** or **E2** as a TFA salt.

To a stirring solution of 4-*N,N*-dimethylamino-1,8-naphthalic (4-DMN) anhydride, DBD-F, or NBD-Cl (0.11 mmol) in dry DMF (1 mL) was added **E1** or **E2** (0.11 mmol) and Et_3N (0.22 mmol) under N_2. The mixture was stirred for 2 h at room temperature (DBD-F, NBD-Cl) or overnight under reflux (4-DMN anhydride). After completion, the reaction was poured into a sat. aq. NH_4Cl solution and extracted with EtOAc. The organic layer was washed with water, brine, and dried over $MgSO_4$. The volatiles were evaporated and the crude product purified by flash silica gel column chromatography (0-20% CH_3OH/CH_2Cl_2) to give probes **1-6**.

1.3 Measurement of ''turn-on'' of fluorescent probe 1.

Probe **1** was prepared into a 10 mM DMSO stock solution and diluted to 1 µM with PBS (pH 7.4) and 150 µM H_2O_2 alone or in presence of 15 µM MPO, 15 µM MPO pre-treated with 500 µM ABAH for 15 min, or 15 µM BSA. The solutions were incubated for 5 min and the fluorescence emission spectra was measured between 480 and 600 nm.

1.4 Probe 1 chemical stability.

Probe **1** was diluted to 400 µM and incubated in CH_3CN, RPMI 1640, or RPMI 1640 supplemented with 2% FCS for 0, 4, and 24 h at 37 °C under constant shaking. At every time point, a 250 µL aliquot was taken. Proteins were precipitated with 750 µL CH_3CN and centrifuged at 15000 x *g* for 15 min. The supernatant was filtered and analyzed by HPLC. To present data as the % of normalized AUC, the AUC for every peak was internally normalized. The normalized AUC for every condition at 0 h was set as 100%.

1.5 Cell culture and differentiation.

HL-60 cells were purchased from Sigma Aldrich (98070106) and were not authenticated. Cells were cultured in RPMI 1640 supplemented with 10% FCS, L-glutamine, and antibiotic-antimycotic solution in a humidified incubator at 37 °C, 5% CO_2 and routinely tested to exclude mycoplasma contamination. Cells were passaged every 2-3 days and only cells passaged no more than 15 times were used for all experiments. To differentiate HL-60 cells into granulocyte-like cells, they were incubated for 5 days with 1.25% DMSO or 1 µM ATRA. After 5 days of differentiation, viability was routinely checked using a trypan blue dye exclusion assay. Differentiation was assessed morphologically by fluorescent nuclear staining (Hoechst 33342) and by evaluation of CD11b expression with a rat primary antibody directed against human CD11b (M1/70, Invitrogen, 1:400, 30 min, 4 °C) using a BD FACSVerse Flow Cytometer. Data were analyzed with FlowJo. For all further studies, the cells were suspended in protein-free RPMI 1640 without phenol red supplemented with L-glutamine.

1.6 NET quantification.

NET quantification was performed as previously described with minimal changes.[1] In brief, cells were plated ($1x10^5$ cells per well) and allowed to settle for 30 min. Subsequently, cells were stimulated with 100 nM PMA or ionomycin 4 µM and incubated for 3 h at 37 °C, 5% CO_2. Unstimulated cells were used as controls. Following incubation, 1 U of micrococcal nuclease was added to detach the DNA from the cell surface and the plate was incubated for 20 min at 37 °C, 5% CO_2. After incubation, the reaction was stopped with 5 mM EDTA and the plates were centrifuged for 10 min at 400 *x g*. Subsequently, the supernatant was collected, 125 nM SYTOX Green was added, and fluorescence measured.

To present data as the % of DNA release and easily analyze the fold changes in NET formation, the data was normalized. Mean fluorescence intensity values from unstimulated cells was set as 100% DNA release. Subsequently, individual fluorescence readouts from each experiment were normalized to this value and shown as NET release (% of unstimulated cells).

1.7 Oxidative burst.

Oxidative burst measurements were performed as previously described with minimal changes[1] using the non-fluorescent dye 2',7'-dichlorodihydrofluorescein diacetate (H2DCFDA), which once oxidized within the cell to 2',7'-dichlorofluorescein (DCF) emits green fluorescence.[33] In brief, cells were loaded with 40 µM H2DCFDA for 30 min at 37 °C, 5% CO_2 in the dark, washed with PBS (pH 7.4), and plated ($1x10^5$ cells per well). Cells were allowed to settle for 30 min and were subsequently stimulated with 100 nM PMA or 4 µM ionomycin. Fluorescence was monitored every 15 min for 3 h.

1.8 Compound characterization data.

***tert*-Butyl (2-((5-carbamoyl-1*H*-imidazol-4-yl)amino)ethyl)carbamate (C1)**

The title compound was obtained as a white crystalline solid (1.14 g, 4.23 mmol, 38% over 2 steps). **^{1}H NMR** (400 MHz, DMSO-*d*$_6$) δ 11.74 (bs, 1H), 7.26 (bs, 1H), 6.88 (t, J = 5.4 Hz, 1H), 6.66 (bs, 2H), 6.06 (bs, 1H), 3.24 (q, J = 6.0 Hz, 2H), 3.06 (q, J = 6.0 Hz, 2H), 1.37 (s, 9H). **^{13}C{^{1}H} NMR** (101 MHz, DMSO-*d*$_6$) δ 155.7, 77.60, 42.61, 40.6, 28.3. **HRMS (ESI)**: *m/z* calcd. for $C_{11}H_{19}N_5O_3$+Na$^+$: 292.1380 [*M*+Na]$^+$; found: 292.1385.

***tert*-Butyl (2-(2-((5-carbamoyl-1*H*-imidazol-4-yl)amino)ethoxy)ethyl)carbamate (C2)**

The title compound was obtained as a white crystalline solid (757.6 mg, 2.42 mmol, 28% over 2 steps). **^{1}H NMR** (400 MHz, DMSO-*d*$_6$) δ 11.67 (bs, 1H), 7.24 (bs, 1H), 6.77 (t, J = 5.9 Hz, 1H), 6.67 (s, 2H), 6.09 (s, 1H), 3.49 (t, J = 5.7 Hz, 2H), 3.38 (t, J = 6.0 Hz, 2H), 3.33 (d, J = 5.7 Hz, 4H), 3.06 (q, J = 6.0 Hz, 2H), 1.36 (s, 9H). **^{13}C{^{1}H} NMR** (101 MHz, DMSO-*d*$_6$) δ 155.6, 77.6, 69.6, 69.1, 43.4, 39.7, 28.2. **HRMS (ESI)**: *m/z* calcd. for $C_{13}H_{23}N_5O_4$+H$^+$: 314.1823 [*M*+H]$^+$; found: 314.1832.

***tert*-Butyl (2-(6-oxo-2-thioxo-1,2,6,7-tetrahydro-3*H*-purin-3-yl)ethyl)carbamate (D1)**

The title compound was obtained as a white solid (350.0 mg, 1.12 mmol, 30% over 2 steps). **^{1}H NMR** (400 MHz, DMSO-*d*$_6$) δ 13.66 (bs, 1H), 12.36 (bs, 1H), 8.13 (s, 1H), 6.79 (t, J = 6.1 Hz, 1H), 4.56 (t, J = 5.8 Hz, 2H), 3.38 (q, J = 5.8 Hz, 2H), 1.28 (s, 9H). **^{13}C{^{1}H} NMR** (101 MHz, DMSO-*d*$_6$) δ 173.8, 155.5, 152.7, 149.8, 141.0, 110.8, 77.4, 47.4, 37.3, 28.1. **HRMS (ESI)**: *m/z* calcd. for $C_{12}H_{17}N_5O_3S$+Na$^+$: 334.0944 [*M*+Na]$^+$; found: 334.0960.

***tert*-Butyl (2-(2-(6-oxo-2-thioxo-1,2,6,7-tetrahydro-3*H*-purin-3-yl)ethoxy)ethyl)carbamate (D2)**

The title compound was obtained as a white solid. **^{1}H NMR** (500 MHz, DMSO-*d*$_6$) δ 13.84 (bs, 1H), 12.47 (bs, 1H), 8.15 (s, 1H), 6.69 (s, 1H), 4.63 (q, J = 5.7 Hz, 2H), 3.76 (q, J = 5.7 Hz, 2H), 3.43 (, J = 5.9 Hz, 2H), 3.01 (p, J = 5.9 Hz, 2H), 1.36 (s, 9H). **^{13}C{^{1}H} NMR** (126 MHz, DMSO-*d*$_6$) δ 173.7, 155.5, 152.5, 149.5, 141.3, 110.6, 77.6, 69.1, 65.7, 46.3, 40.0, 28.2. **HRMS (ESI)**: *m/z* calcd. for $C_{14}H_{21}N_5O_4S$+Na$^+$: 378.1206 [*M*+Na]$^+$; found: 378.1216.

3-(2-Aminoethyl)-2-thioxo-1,2,3,7-tetrahydro-6*H*-purin-6-one, TFA salt (E1)

The title compound was obtained as a white powder (123 mg, 0.38 mmol, 97%). **^{1}H NMR** (400 MHz, DMSO-*d*$_6$) δ 13.95 (s, 1H), 12.57 (s, 1H), 8.22 (s, 1H), 7.91 (s, 3H), 4.75 (t, J = 6.1 Hz, 2H), 3.28 (s, 2H). **^{13}C{^{1}H} NMR** (101 MHz, DMSO-*d*$_6$) δ 174.1, 152.7, 149.5, 141.3, 111.0, 44.9, 36.8. **HRMS (ESI)**: *m/z* calcd. for $C_7H_9N_5OS$+H$^+$: 212.0601 [*M*+H]$^+$; found: 212.0597.

3-(2-(2-Aminoethoxy)ethyl)-2-thioxo-1,2,3,7-tetrahydro-6*H*-purin-6-one, TFA salt (E2)

The title compound was obtained as a white powder (64.3 mg, 0.17 mmol, 49%). **^{1}H NMR** (500 MHz, DMSO-*d*$_6$) δ 13.91 (s, 1H), 12.51 (s, 1H), 8.18 (s, 1H), 7.77 (s, 3H), 4.69 (t, J = 6.4 Hz, 2H), 3.86 (t, J = 6.4 Hz, 2H), 3.68 (t, J = 5.3 Hz, 2H), 2.95 (q, J = 5.4 Hz, 2H). **^{13}C{^{1}H} NMR** (126 MHz, DMSO-*d*$_6$) δ 173.7, 152.6, 149.5, 141.4, 110.7, 66.5, 66.0, 46.1, 38.7. **HRMS (ESI)**: *m/z* calcd. for $C_9H_{13}N_5O_2S$+H$^+$: 256.0863 [*M*+H]$^+$; found: 256.0856.

6-(Dimethylamino)-2-(2-(6-oxo-2-thioxo-1,2,6,7-tetrahydro-3*H*-purin-3-yl)ethyl)-1H-benzo[de]isoquinoline-1,3(2*H*)-dione (1)

The title compound was obtained as an orange powder (15.0 mg, 0.03 mmol, 60%). **^{1}H NMR** (400 MHz, DMSO-*d*$_6$) δ 13.63 (s, 1H), 12.27 (s, 1H), 8.47 (dd, J = 8.5, 1.2 Hz, 1H), 8.34 (dd, J = 7.2, 1.1 Hz, 1H), 8.22 (d, J = 8.3 Hz, 1H), 7.83 (s, 1H), 7.70 (dd, J = 8.5, 7.2 Hz, 1H), 7.16 (d, J = 8.3 Hz, 1H), 5.02 – 4.82 (m, 2H), 4.62 – 4.46 (m, 2H), 3.07 (s, 6H). **^{13}C{^{1}H} NMR** (101 MHz, DMSO-*d*$_6$) δ 173.9, 163.9, 163.1, 156.2, 152.3, 149.5, 141.4, 131.8, 131.1, 130.1, 129.5, 124.7, 124.0, 122.1, 113.2, 112.7, 110.3, 45.7, 44.2, 37.4. **HPLC** (linear gradient 10 → 90% CH_3CN in 0.1% TFA in Milli-Q, 10 min): R_t (min): 5.14. **HRMS (ESI)**: *m/z* calcd. for $C_{21}H_{18}N_6O_3S$+H$^+$: 435.1234 [*M*+H]$^+$; found: 435.1229.

6-(Dimethylamino)-2-(2-(2-(6-oxo-2-thioxo-1,2,6,7-tetrahydro-3*H*-purin-3-yl)ethoxy)ethyl)-1*H*-benzo[de]isoquinoline-1,3(2*H*)-dione (2)

The title compound was obtained as an orange powder (20.0 mg, 0.04 mmol, 70%). **^{1}H NMR** (500 MHz, DMSO-*d*$_6$) δ 12.24 (bs, 1H), 8.51 (dd, J = 8.5, 1.2 Hz, 1H), 8.43 (dd, J = 7.3, 1.2 Hz, 1H), 8.32 (d, J = 8.2 Hz, 1H), 7.89 (s, 1H), 7.75 (dd, J = 8.5, 7.3 Hz, 1H), 7.21 (d, J = 8.2 Hz, 1H), 4.60 (t, J = 6.5 Hz, 2H), 4.18 (t, J = 6.3 Hz, 2H), 3.81 (t, J = 6.5 Hz, 2H), 3.69 (t, J = 6.3 Hz, 2H), 3.10 (s, 6H). **^{13}C{^{1}H} NMR** (101 MHz, DMSO-*d*$_6$) δ 174.1, 164.1, 163.4, 156.4, 152.5, 149.7, 141.7, 132.0, 131.3, 130.3, 129.7, 124.9, 124.2, 122.3, 113.4, 113.0, 110.5, 45.9, 44.4, 37.6. **HPLC** (linear gradient 10 → 90% CH_3CN in 0.1% TFA in Milli-Q, 10 min): R_t (min): 5.21. **HRMS (ESI)**: *m/z* calcd. for $C_{23}H_{22}N_6O_4S$+H$^+$: 479.1496 [*M*+H]$^+$; found: 479.1509.

***N,N*-Dimethyl-7-((2-(6-oxo-2-thioxo-1,2,6,7-tetrahydro-3*H*-purin-3-yl)ethyl)amino)benzo[c][1,2,5]oxadiazole-4-sulfonamide (3)**

The title compound was obtained as a yellow powder (13.7 mg, 0.03 mmol, 67%). **^{1}H NMR** (400 MHz, DMSO-*d*$_6$) δ 13.79 (s, 1H), 12.49 (s, 1H), 8.44 (t, J = 6.2 Hz, 1H), 8.06 (s, 1H), 7.81 (d, J = 8.2 Hz, 1H), 6.57 (d, J = 8.2 Hz, 1H), 4.80 (t, J = 6.2 Hz, 2H), 3.86 (q, J = 6.2 Hz, 2H), 2.68 (s, 6H). **^{13}C{^{1}H} NMR** (126 MHz, DMSO-*d*$_6$) δ 174.1, 152.8, 149.7, 146.6, 144.5, 141.8, 141.4, 140.4, 111.0, 105.7, 99.4, 45.7, 40.1, 37.7. **HPLC** (linear gradient 10 → 90% CH_3CN in 0.1% TFA in Milli-Q, 10 min): R_t (min): 4.81. **HRMS (ESI)**: *m/z* calcd. for $C_{15}H_{16}N_8O_4S_2$+H$^+$: 437.0809 [*M*+H]$^+$; found: 437.0825.

_N,N_-Dimethyl-7-((2-(2-(6-oxo-2-thioxo-1,2,6,7-tetrahydro-3_H_-purin-3-yl)ethoxy)ethyl)amino)benzo[c][1,2,5]oxadiazole-4-sulfonamide (4)

The title compound was obtained as a yellow powder (17.7 mg, 0.04 mmol, 88%). **^{1}H NMR** (400 MHz, DMSO-d_6) δ 13.83 (s, 1H), 12.47 (s, 1H), 8.24 (t, J = 5.8 Hz, 1H), 8.12 (s, 1H), 7.80 (d, J = 8.2 Hz, 1H), 6.35 (d, J = 8.2 Hz, 1H), 4.66 (t, J = 6.3 Hz, 2H), 3.85 (t, J = 6.3 Hz, 2H), 3.75 (t, J = 5.8 Hz, 2H), 3.51 (t, J = 5.8 Hz, 2H), 2.69 (s, 6H). **^{13}C{^{1}H} NMR** (101 MHz, DMSO-d_6) δ 173.7, 152.5, 149.5, 146.5, 144.3, 141.4, 141.3, 140.4, 110.7, 105.2, 99.0, 68.0, 66.0, 46.3, 42.9, 37.5. **HPLC** (linear gradient 10 → 90% CH_3CN in 0.1% TFA in Milli-Q, 10 min): R_t (min): 4.84. **HRMS (ESI)**: *m/z* calcd. for $C_{17}H_{20}N_8O_5S_2+H^+$: 481.1071 [*M*+H]$^+$; found: 481.1073.

3-(2-((7-Nitrobenzo[c][1,2,5]oxadiazol-4-yl)amino)ethyl)-2-thioxo-1,2,3,7-tetrahydro-6_H_-purin-6-one (5)

The title compound was obtained as a brown-green powder (18.0 mg, 0.05 mmol, 79%). **^{1}H NMR** (500 MHz, DMSO-d_6) δ 13.77 (s, 1H), 12.51 (s, 1H), 9.47 (t, J = 6.4 Hz, 1H), 8.56 (d, J = 9.0 Hz, 1H), 8.00 (s, 1H), 6.66 (d, J = 9.0 Hz, 1H), 4.81 (t, J = 6.4 Hz, 2H), 3.97 (t, J = 6.4 Hz, 2H). **^{13}C{^{1}H} NMR** (126 MHz, DMSO-d_6) δ 173.9, 152.6, 149.5, 145.6, 144.5, 144.0, 141.2, 137.8, 121.1, 110.8, 99.6, 45.3, 40.3. **HPLC** (linear gradient 10 → 90% CH_3CN in 0.1% TFA in Milli-Q, 10 min): R_t (min): 4.64. **HRMS (ESI)**: *m/z* calcd. for $C_{13}H_{10}N_8O_4S+Na^+$: 397.0438 [*M*+Na]$^+$; found: 397.0447.

3-(2-(2-((7-Nitrobenzo[c][1,2,5]oxadiazol-4-yl)amino)ethoxy)ethyl)-2-thioxo-1,2,3,7-tetrahydro-6_H_-purin-6-one (6)

The title compound was obtained as a brown-green powder (17.5 mg, 0.04 mmol, 87%). **^{1}H NMR** (400 MHz, DMSO-d_6) δ 13.77 (s, 1H), 12.43 (s, 1H), 9.37 (s, 1H), 8.47 (d, J = 9.0 Hz, 1H), 8.06 (s, 1H), 6.41 (d, J = 9.0 Hz, 1H), 4.64 (t, J = 6.2 Hz, 2H), 3.87 (t, J = 6.2 Hz, 2H), 3.77 (t, J = 5.3 Hz, 2H), 3.63 – 3.54 (m, 2H). **^{13}C{^{1}H} NMR** (126 MHz, DMSO-d_6) δ 173.6, 152.4, 149.5, 145.3, 144.3, 141.2, 140.0, 137.8, 120.9, 110.6, 99.4, 68.0, 66.0, 46.3, 43.3 **HPLC** (linear gradient 10 → 90% CH_3CN in 0.1% TFA in Milli-Q, 10 min): R_t (min): 4.43. **HRMS (ESI)**: *m/z* calcd. for $C_{15}H_{14}N_8O_5S+Na^+$: 441.0700 [*M*+Na]$^+$; found: 441.0719.

2. Supplementary Tables.

Supplementary Table 1. Spectral properties of probes **1-6** in CH_2CH_2, CH_3CN, CH_3OH, and PBS (pH 7.4).

Probe	Solvent	λ_{ex} (nm)	λ_{em} (nm)	SS[a] (nm)	ε ($M^{-1}cm^{-1}$)
**1**	CH_2Cl_2	415	510	95	
	CH_3CN	411	529	118	
	CH_3OH	427	533	106	
	PBS (pH 7.4)	457	560	103	7200
**2**	CH_2Cl_2	415	512	97	
	CH_3CN	415	532	117	
	CH_3OH	434	540	106	
	PBS (pH 7.4)	446	560	114	6500
**3**	CH_2Cl_2	417	532	115	
	CH_3CN	426	552	126	
	CH_3OH	431	570	139	
	PBS (pH 7.4)	435	600	165	6200
**4**	CH_2Cl_2	420	535	115	
	CH_3CN	428	560	132	
	CH_3OH	430	578	148	
	PBS (pH 7.4)	435	600	165	5900
**5**	CH_2Cl_2	452	522	70	
	CH_3CN	458	533	75	
	CH_3OH	460	535	75	
	PBS (pH 7.4)	473	558	85	12700
**6**	CH_2Cl_2	452	517	65	
	CH_3CN	460	531	71	
	CH_3OH	465	534	69	
	PBS (pH 7.4)	488	558	70	14800

[a]SS, Stokes shift.

3. Supplementary Figures.

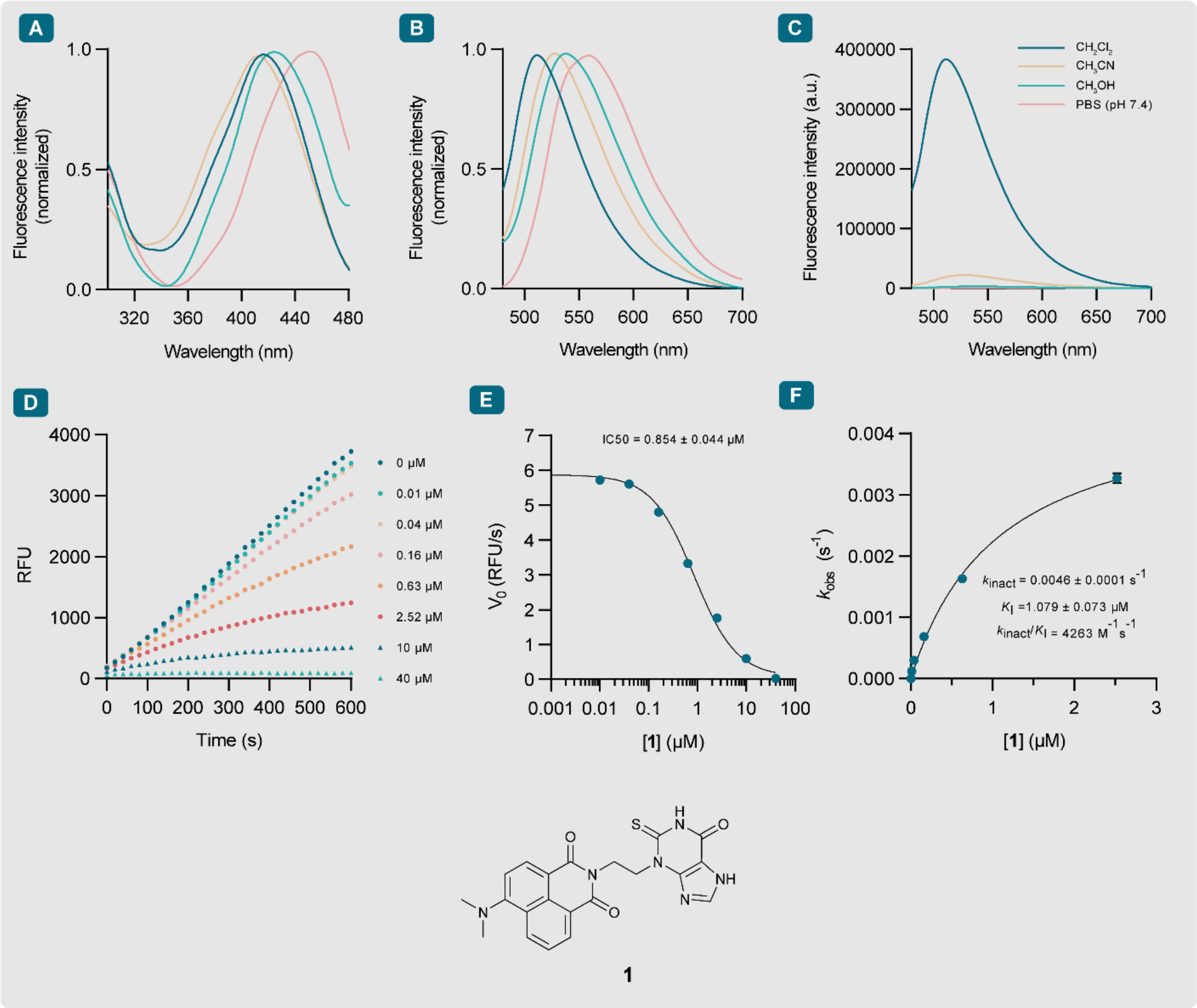

Supplementary Figure 1. Spectroscopic and functional properties of probe 1. (A) Normalized fluorescence excitation spectrum in CH_2Cl_2, CH_3CN, CH_3OH, PBS (pH 7.4). (B) Normalized fluorescence emission spectrum. (C) Fluorescence emission spectrum. Probe **1** displays a x315 fluorescence increase at λ_{em} in CH_2Cl_2 compared to PBS (pH 7.4). (D) MPO inhibition progress curve with probe **1** concentrations ranging from 0.01 to 40 µM. (E) Initial velocities (V_0) from (D) were plotted against probe **1** concentration and fit to a dose-response curve with a Hill slope of -1 to determine the relative IC_{50} value. (F) The k_{obs} values were plotted against probe **1** concentration and the k_{inact}, K_I, and k_{inact}/K_I values were calculated. The data is presented as mean ± SD (n = 5 technical replicates).

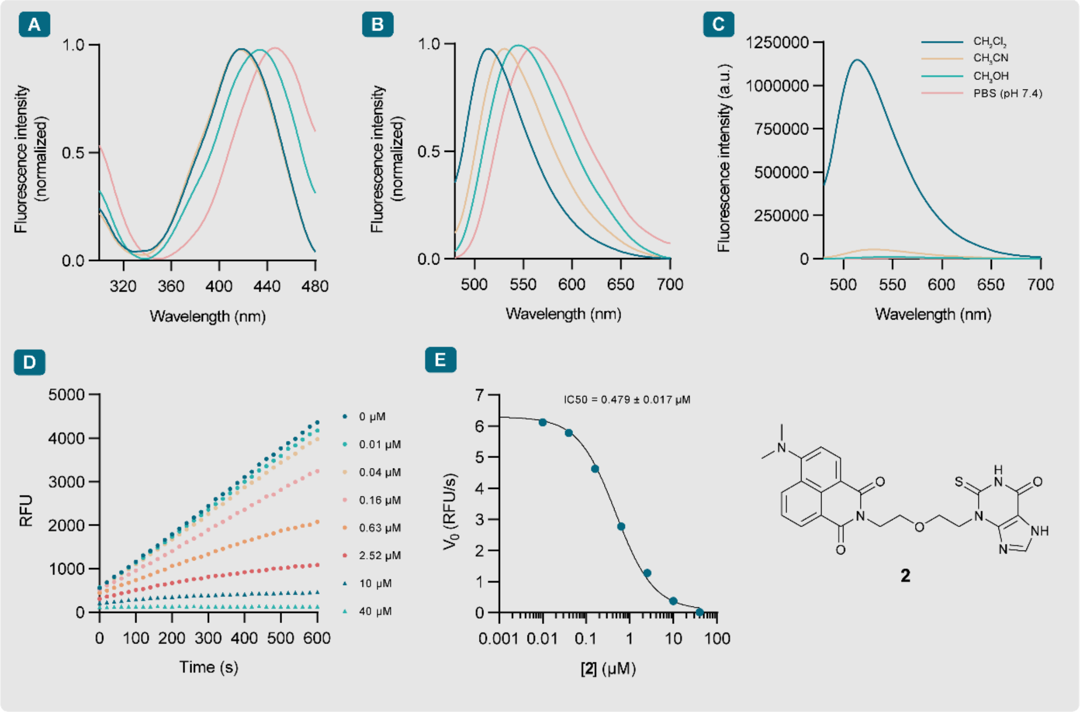

Supplementary Figure 2. Spectroscopic and functional properties of probe 2. (A) Normalized fluorescence excitation spectrum in CH_2Cl_2, CH_3CN, CH_3OH, PBS (pH 7.4). (B) Normalized fluorescence emission spectrum. (C) Fluorescence emission spectrum. Probe **2** displays a x283 fluorescence increase at λ_{em} in CH_2Cl_2 compared to PBS (pH 7.4). (D) MPO inhibition progress curve with probe **2** concentrations ranging from 0.01 to 40 µM. (E) Initial velocities (V_0) from (D) were plotted against probe **2** concentration and fit to a dose-response curve with a Hill slope of -1 to determine the relative IC_{50} value. The data is presented as mean ± SD (n = 5 technical replicates).

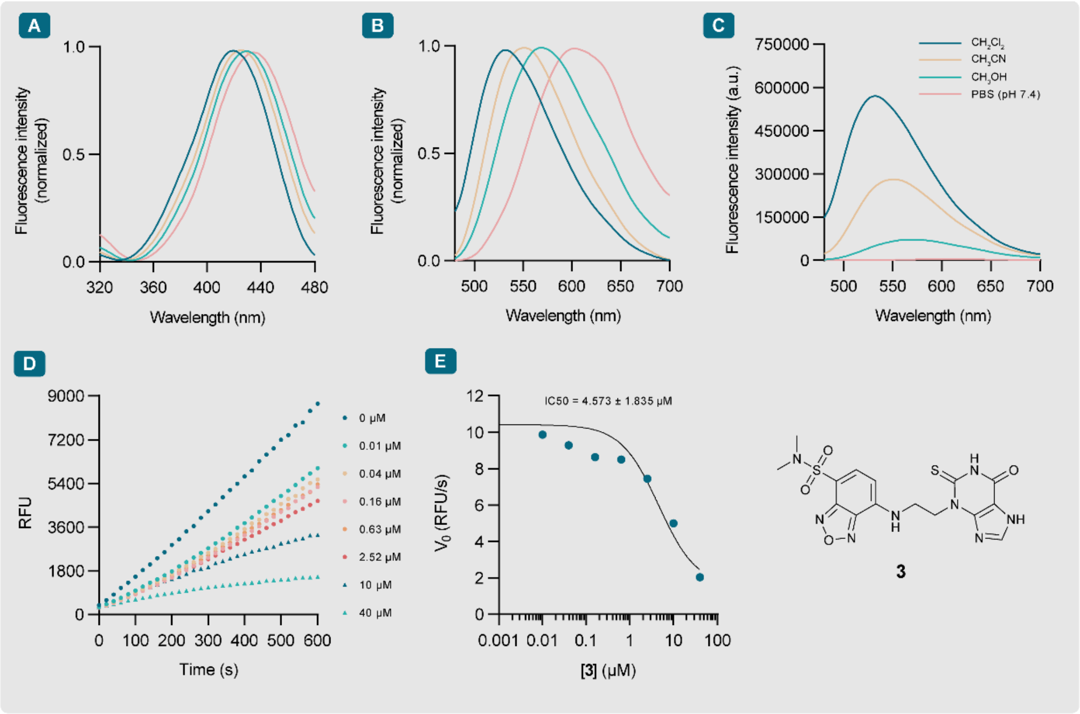

Supplementary Figure 3. Spectroscopic and functional properties of probe 3. (A) Normalized fluorescence excitation spectrum in CH_2Cl_2, CH_3CN, CH_3OH, PBS (pH 7.4). (B) Normalized fluorescence emission spectrum. (C) Fluorescence emission spectrum. Probe **3** displays a x283 fluorescence increase at λ_{em} in CH_2Cl_2 compared to PBS (pH 7.4). (D) MPO inhibition progress curve with probe **3** concentrations ranging from 0.01 to 40 µM. (E) Initial velocities (V_0) from (D) were plotted against probe **3** concentration and fit to a dose-response curve with a Hill slope of -1 to determine the relative IC_{50} value. The data is presented as mean ± SD (n = 5 technical replicates).

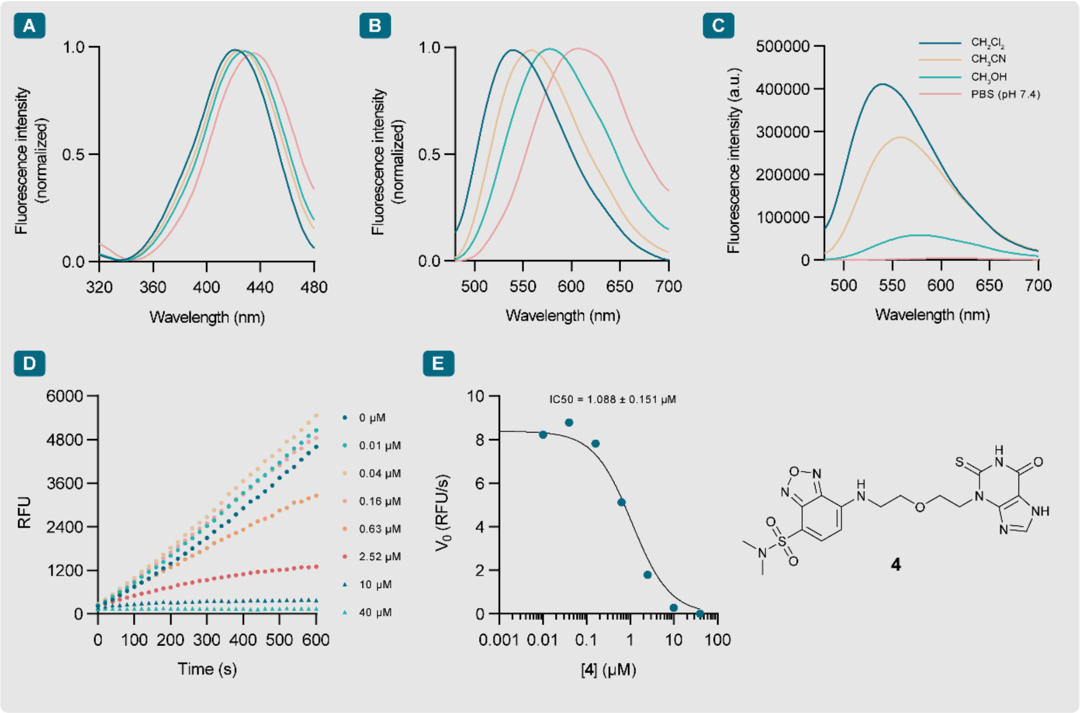

Supplementary Figure 4. Spectroscopic and functional properties of probe 4. (A) Normalized fluorescence excitation spectrum in CH_2Cl_2, CH_3CN, CH_3OH, PBS (pH 7.4). (B) Normalized fluorescence emission spectrum. (C) Fluorescence emission spectrum. Probe **4** displays a x99 fluorescence increase at λ_{em} in CH_2Cl_2 compared to PBS (pH 7.4). (D) MPO inhibition progress curve with probe **4** concentrations ranging from 0.01 to 40 µM. (E) Initial velocities (V_0) from (D) were plotted against probe **4** concentration and fit to a dose-response curve with a Hill slope of -1 to determine the relative IC_{50} value. The data is presented as mean ± SD (n = 5 technical replicates).

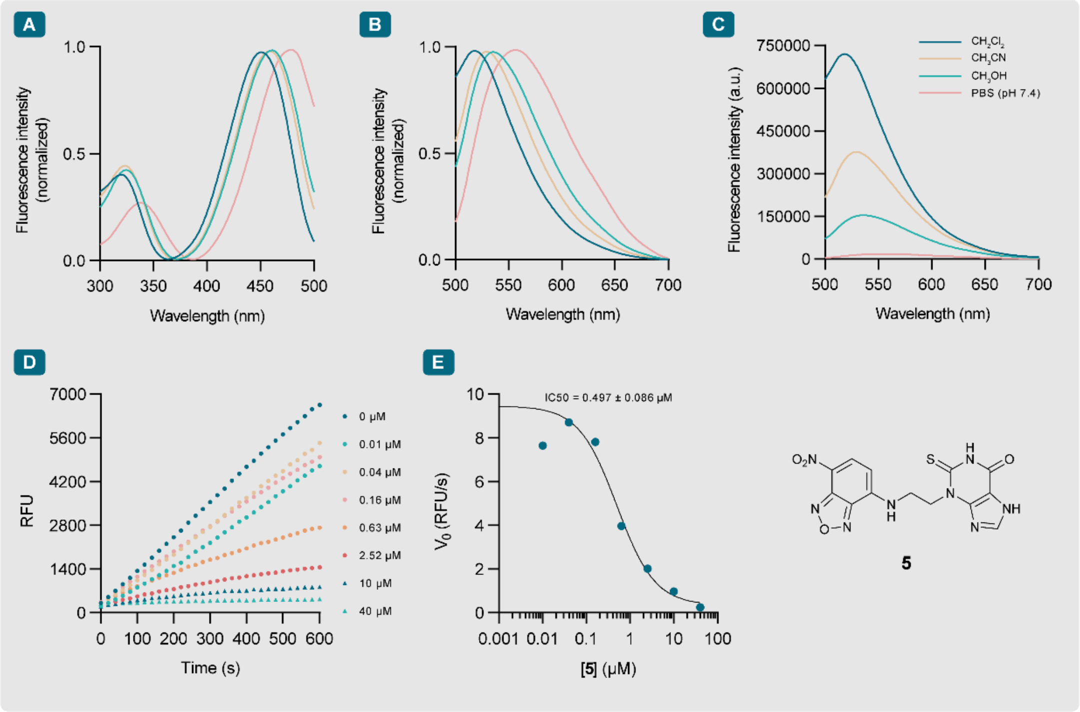

Supplementary Figure 5. Spectroscopic and functional properties of probe 5. (A) Normalized fluorescence excitation spectrum in CH_2Cl_2, CH_3CN, CH_3OH, PBS (pH 7.4). (B) Normalized fluorescence emission spectrum. (C) Fluorescence emission spectrum. Probe **5** displays a x40 fluorescence increase at λ_{em} in CH_2Cl_2 compared to PBS (pH 7.4). (D) MPO inhibition progress curve with probe **5** concentrations ranging from 0.01 to 40 µM. (E) Initial velocities (V_0) from (D) were plotted against probe **5** concentration and fit to a dose-response curve with a Hill slope of -1 to determine the relative IC_{50} value. The data is presented as mean ± SD (n = 5 technical replicates).

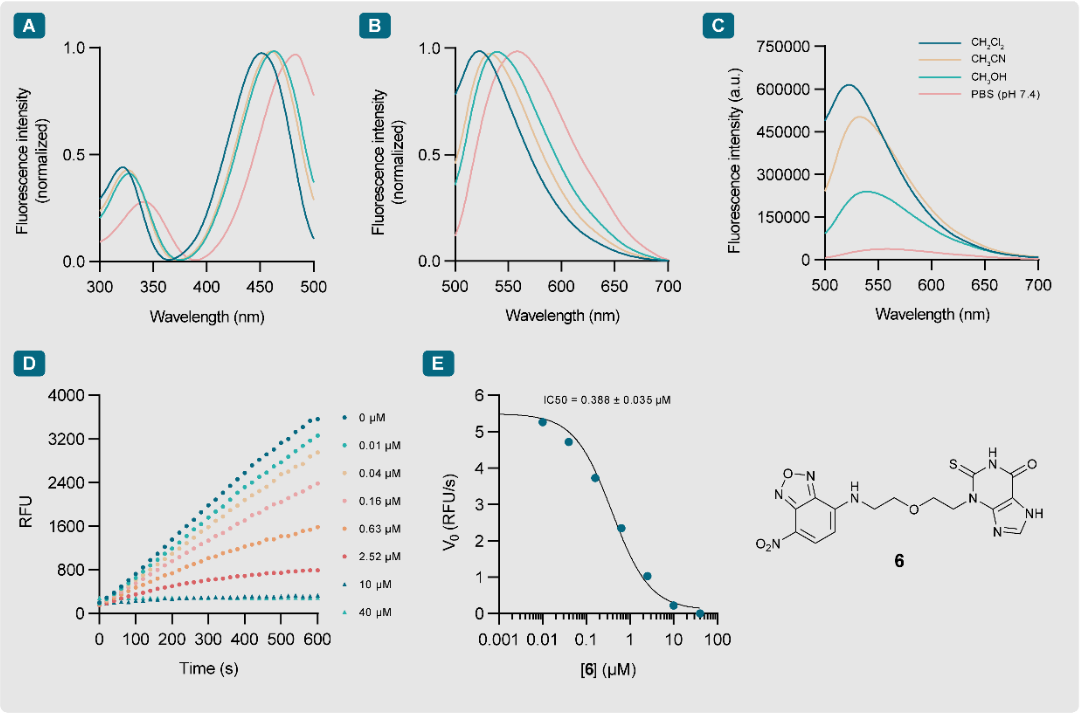

Supplementary Figure 6. Spectroscopic and functional properties of probe 6. (A) Normalized fluorescence excitation spectrum in CH_2Cl_2, CH_3CN, CH_3OH, PBS (pH 7.4). (B) Normalized fluorescence emission spectrum. (C) Fluorescence emission spectrum. Probe **6** displays a x16 fluorescence increase at λ_{em} in CH_2Cl_2 compared to PBS (pH 7.4). (D) MPO inhibition progress curve with probe **6** concentrations ranging from 0.01 to 40 µM. (E) Initial velocities (V_0) from (D) were plotted against probe **6** concentration and fit to a dose-response curve with a Hill slope of -1 to determine the relative IC_{50} value. The data is presented as mean ± SD (n = 4 technical replicates).

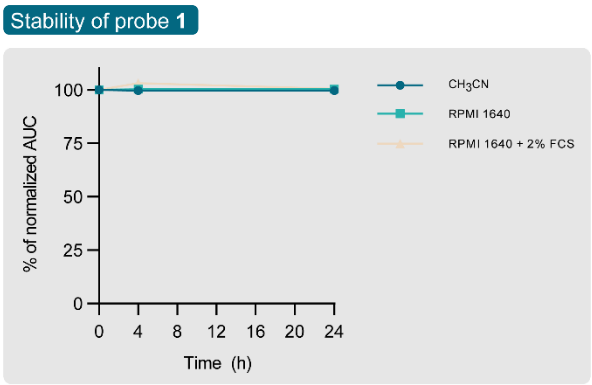

Supplementary Figure 7. Chemical stability of probe 1. Chemical stability of probe **1** was measured at 400 µM in CH_3CN, RPMI 1640, and RPMI 1640 + 2% FCS at 0, 4, and 24 h. The data is presented as percentage of the normalized AUC of probe **1** at 0 h measured by HPLC.

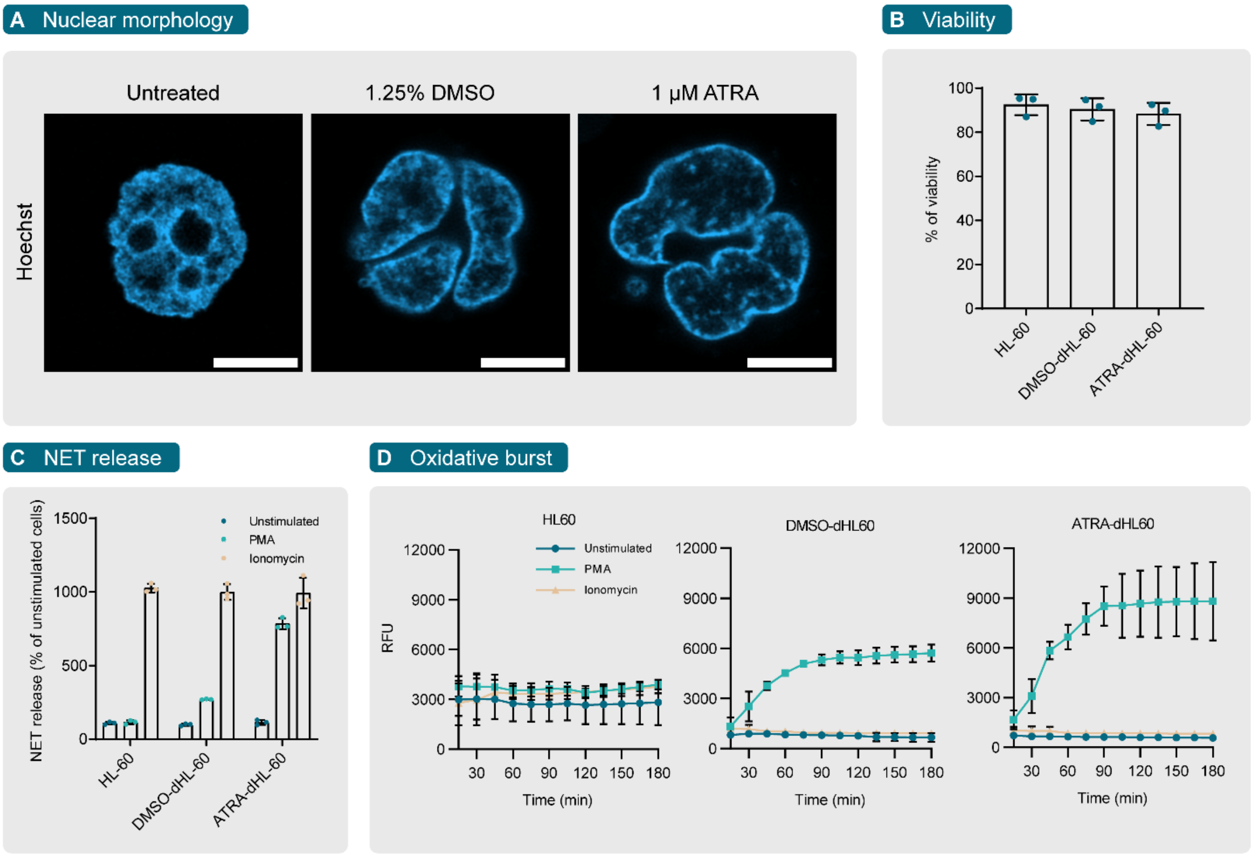

Supplementary Figure 8. Differentiation of HL-60 cells into dHL-60 granulocytes. (A) Nuclear morphology of untreated HL-60 cells or HL-60 cells treated with 1.25% DMSO or 1 µM ATRA for 5 days. Scale bar = 5 µm. (B) Viability of control HL-60 cells or HL-60 cells after treatment (n = 3 independent experiments). (C) NET release quantification in HL-60 cells or HL-60-derived granulocytes stimulated with 100 nM PMA or 4 µM ionomycin for 3 h. Data presented as % of unstimulated cells (n = 3 technical replicates). (D) Oxidative burst of HL-60 or HL-60-derived granulocytes loaded with H_2DCFDA and stimulated with 100 nM PMA or 4 µM ionomycin for 3 h (n = 3 technical replicates). The data is presented as mean ± SD.

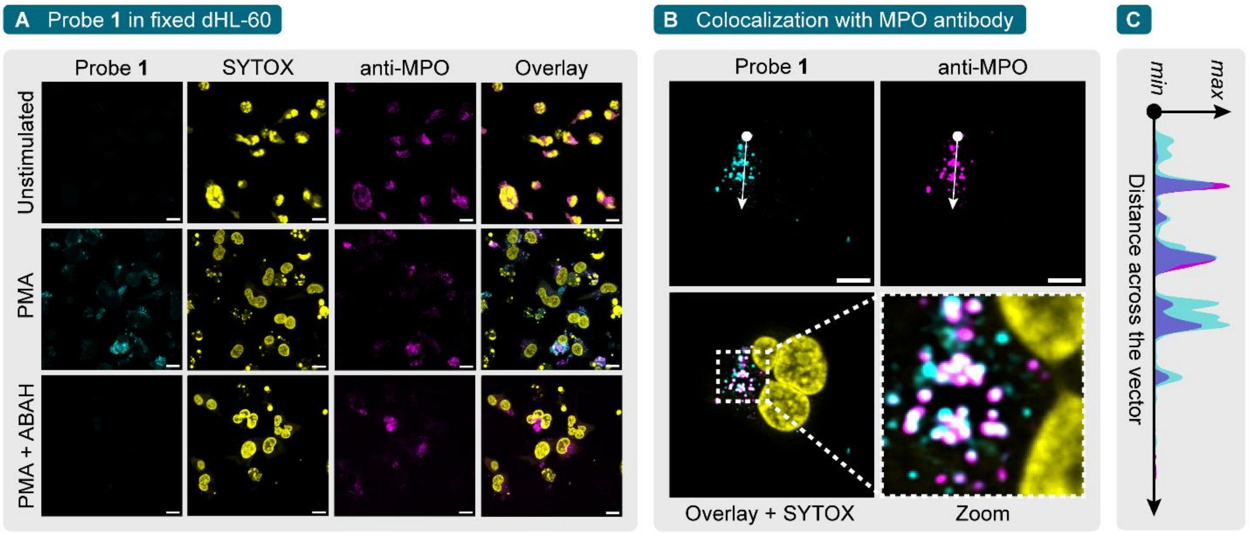

Supplementary Figure 9. Qualitative colocalization of probe 1 with MPO in dHL-60 granulocytes. (A) CLSM panel of fixed dHL-60 cells stimulated with PMA in the presence of **1** µM probe 1 (cyan) for 3 h at 37 °C, 5% CO2. Where appropriate, cells were pre-treated with 100 µM ABAH for 30 min before the addition of probe 1. NETs were detected with SYTOX Orange (yellow). MPO was detected by immunostaining with a primary anti-human MPO antibody and a secondary goat-anti human antibody conjugated to AF647 (magenta). (B) Colocalization of probe **1** and anti-human MPO antibody in hPMN stimulated with PMA. (C) Histogram overlap (purple) of probe **1** (cyan) and anti-MPO antibody (magenta) normalized intensity as a function of the distance across the vector in (B). Scale bar = 10 µm.

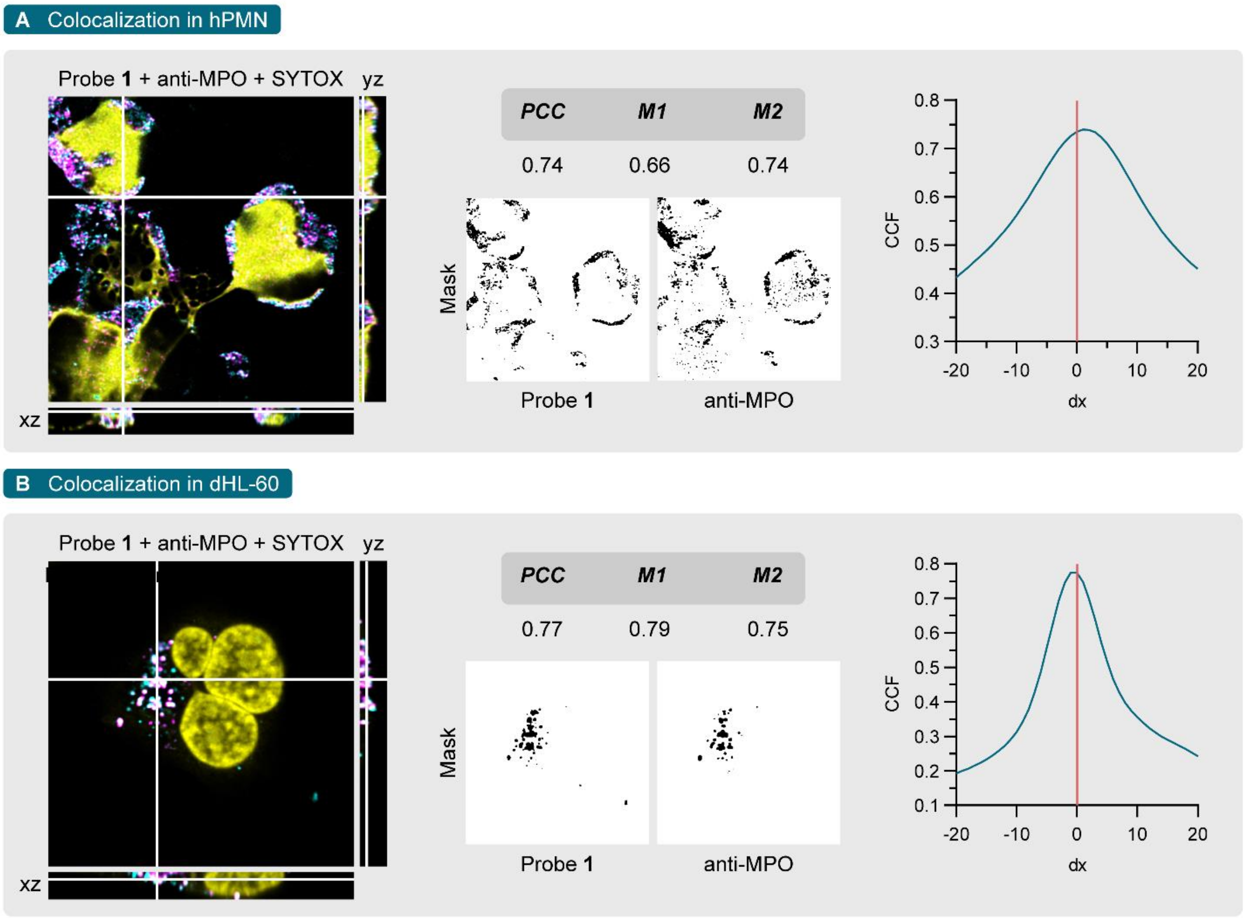

Supplementary Figure 10. Quantitative colocalization of probe 1 with MPO in hPMN and dHL-60 granulocytes. (A) Colocalization assessment and quantification of probe **1** and anti-MPO antibody in hPMN. PCC, M1 and M2, and Van Steensel's CFF curve were obtained by analysing a 2.6 µm Z-stack using the exemplary mask panel. (B) Colocalization assessment and quantification in dHL-60 cells.

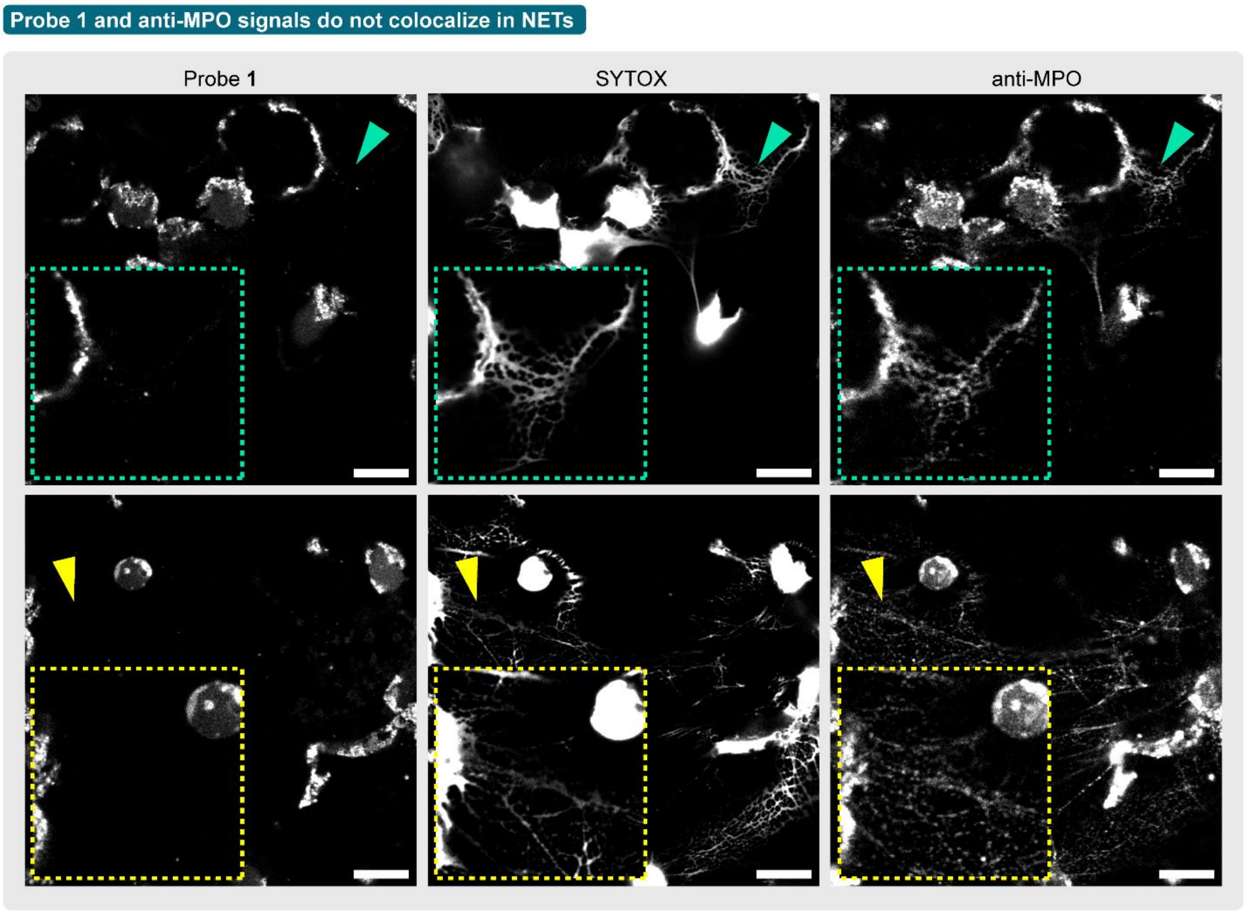

Supplementary Figure 11. Probe 1 and anti-MPO signals do not colocalize in NETs of PMA-stimulated hPMNs. DNA was stained with SYTOX Orange. Anti-MPO antibody, but not probe **1,** reports the presence of MPO in NETs. Panel rows represent two independent experiments. Scale bar = 10 µm.

5. Supplementary References.

1. Manda-Handzlik A, Bystrzycka W, Wachowska M, Sieczkowska S, Stelmaszczyk-Emmel A, Demkow U, et al. The influence of agents differentiating HL-60 cells toward granulocyte-like cells on their ability to release neutrophil extracellular traps. Immunology & Cell Biology. 2018;96(4):413–25.
