# Supplementary material for: Environment-sensitive turn-on fluorescent probe enables live cell imaging of myeloperoxidase activity during NETosis
Source: Commun Chem. 2024 Nov 12;7:262. doi: 10.1038/s42004-024-01338-5 (PMC11557929; doi:10.1038/s42004-024-01338-5)
Supplement: Supplementary file 3 — Description of Additional Supplementary Files [file 42004_2024_1338_MOESM3_ESM.pdf]

Description of Additional Supplementary Files

File name: Supplementary Data 1

Description: NMR spectra of synthetic intermediates and probes 1-6.

File name: Supplementary Data 2

Description: HPLC traces of probes 1-6.
