# Supplementary material for: Environment-sensitive turn-on fluorescent probe enables live cell imaging of myeloperoxidase activity during NETosis
Source: Commun Chem. 2024 Nov 12;7:262. doi: 10.1038/s42004-024-01338-5 (PMC11557929; doi:10.1038/s42004-024-01338-5)
Supplement: Supplementary file 4 — Supplementary Data 1 [file 42004_2024_1338_MOESM4_ESM.docx]

# 1. Supplementary Data 1.

# 1.1 NMR spectra.

**
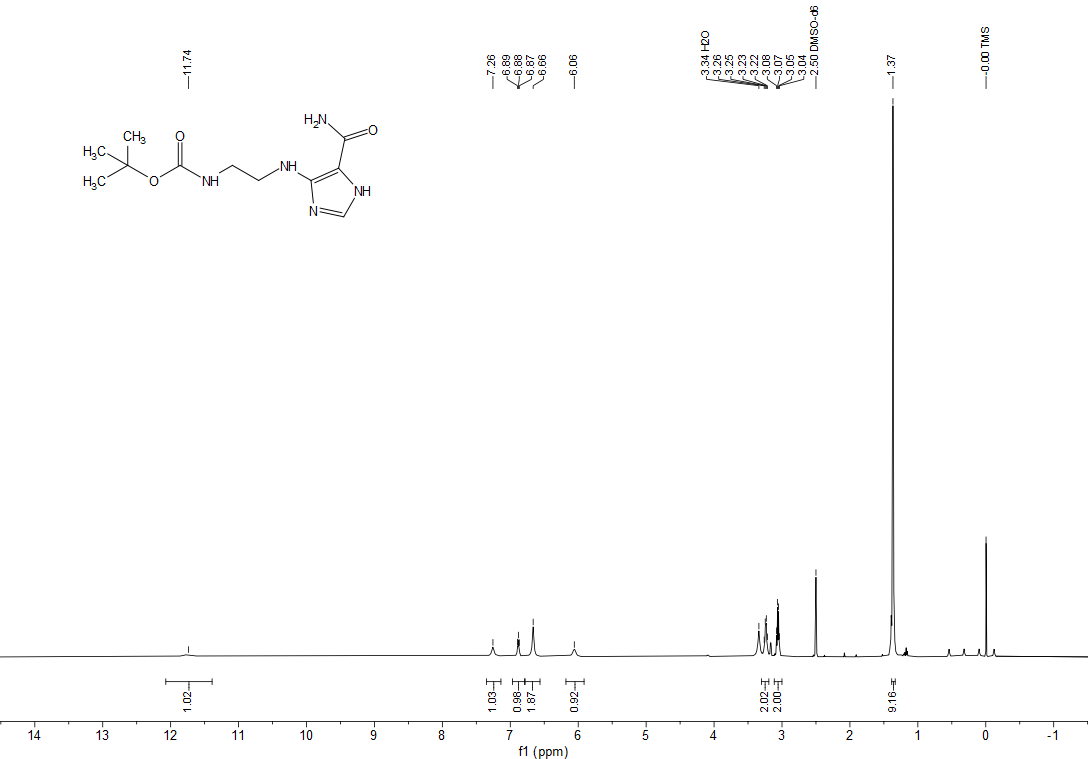
**

^1^H NMR (400 MHz, DMSO-*d*_6_) of **C1**.

**
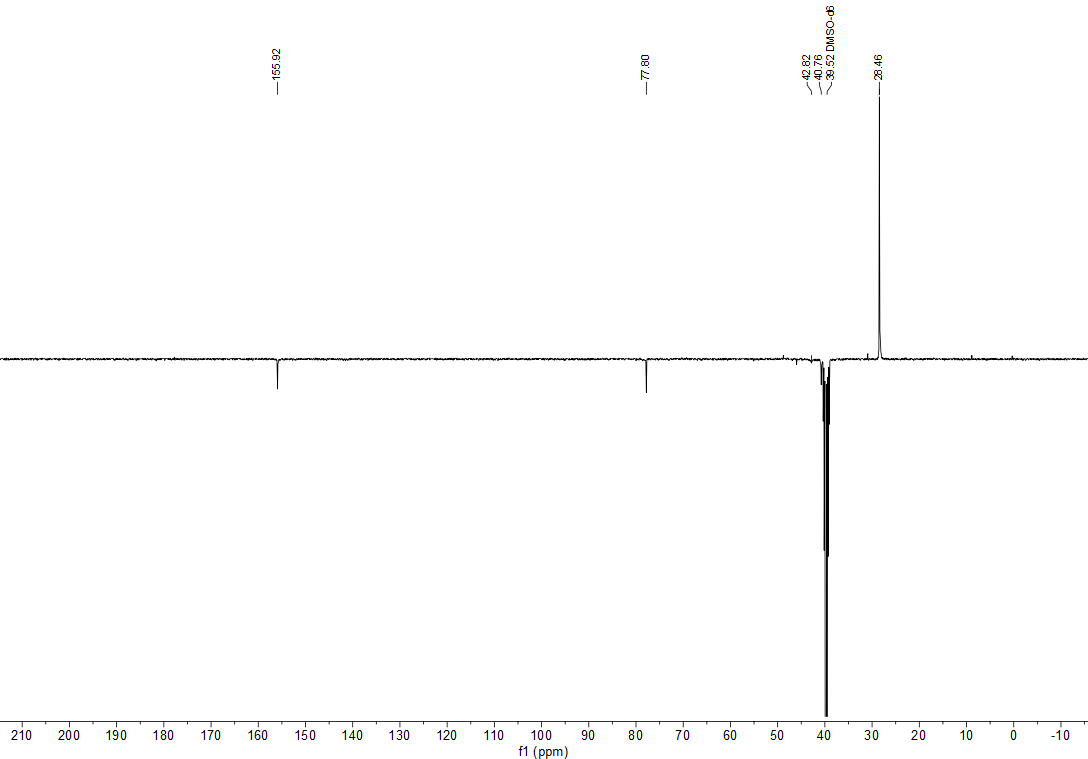
**

^13^C{^1^H} NMR (101 MHz, DMSO-*d*_6_, APT) of **C1**. Imidazole ^13^C signals could not be detected. The signals were present in the compounds that followed.

**
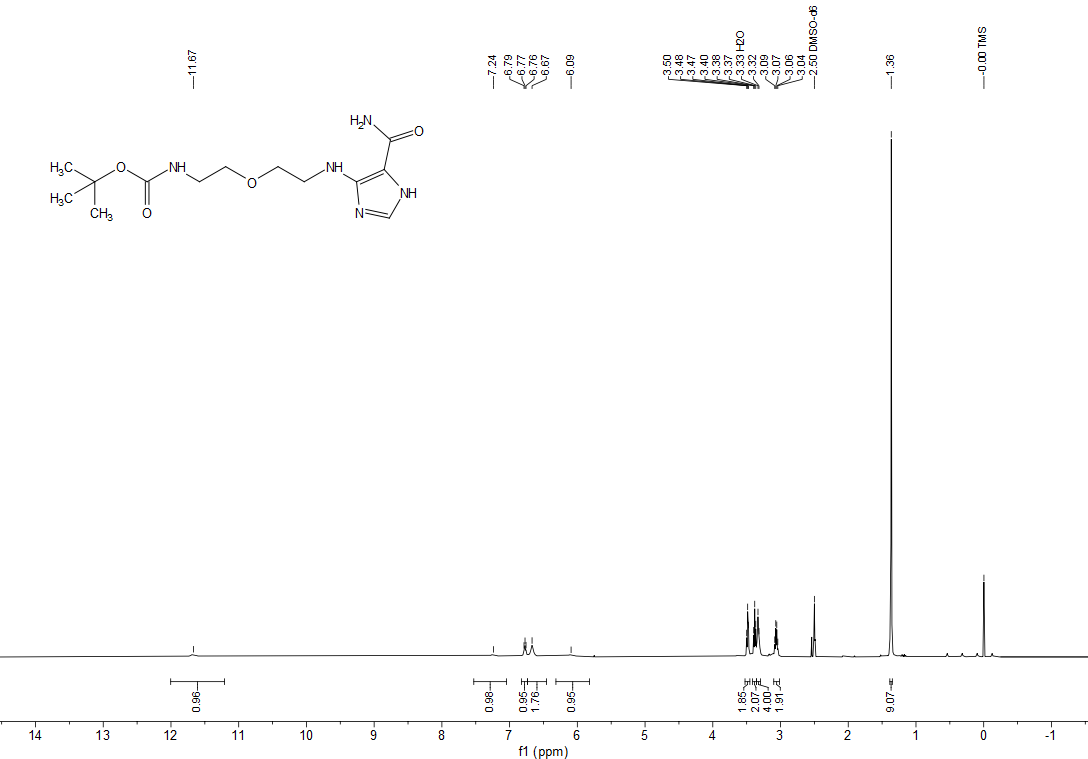
**

^1^H NMR (400 MHz, DMSO-*d*_6_) of **C2**.


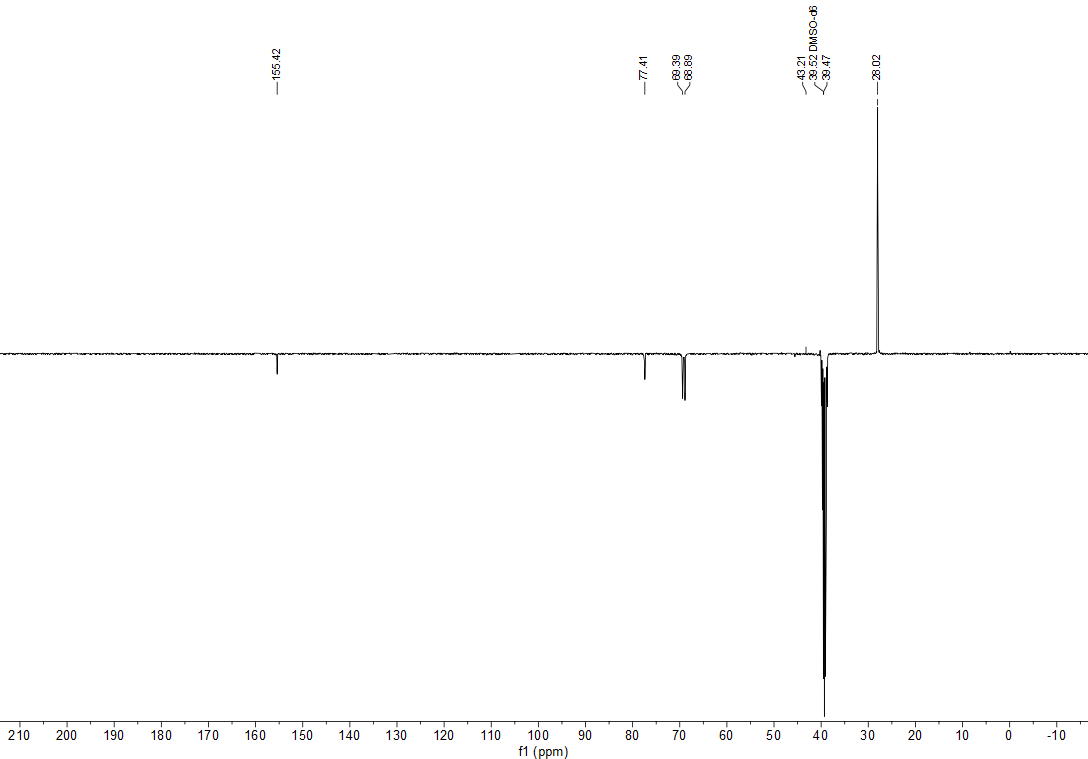


^13^C{^1^H} NMR (101 MHz, DMSO-*d*_6_, APT) of **C2**. Imidazole ^13^C signals could not be detected. The signals were present in the compounds that followed.


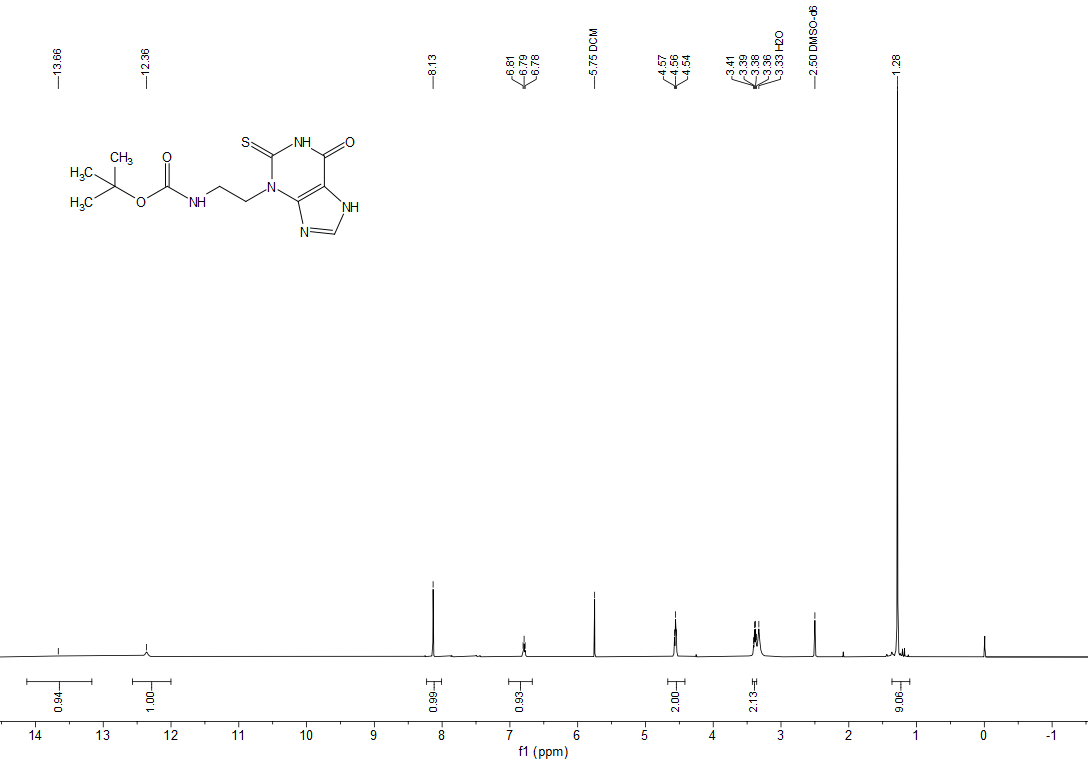


^1^H NMR (400 MHz, DMSO-*d*_6_) of **D1**.


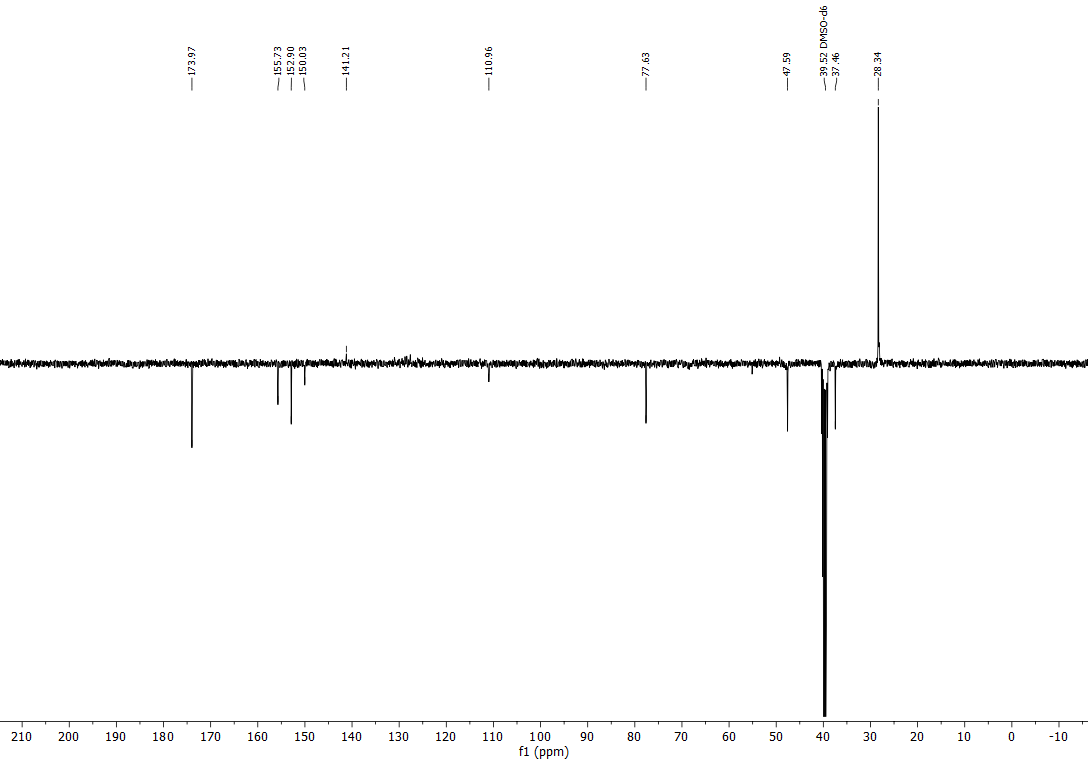


^13^C{^1^H} NMR (101 MHz, DMSO-*d*_6_, APT) of **D1**.


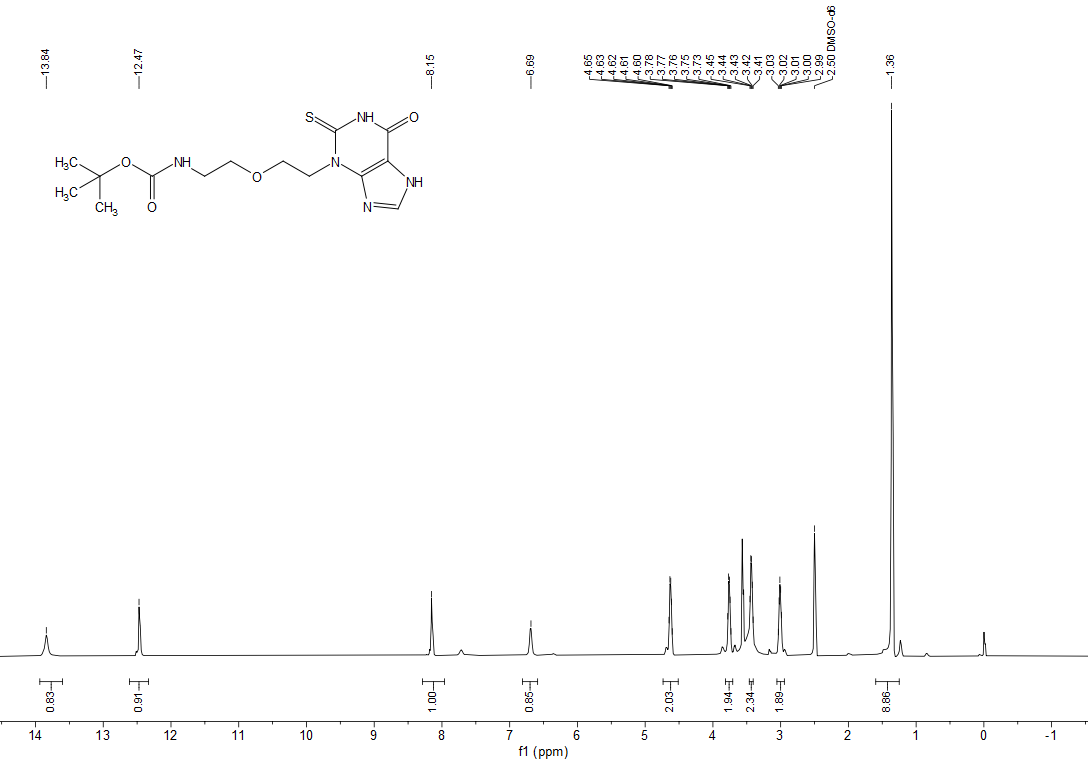


^1^H NMR (500 MHz, DMSO-*d*_6_) of **D2**.


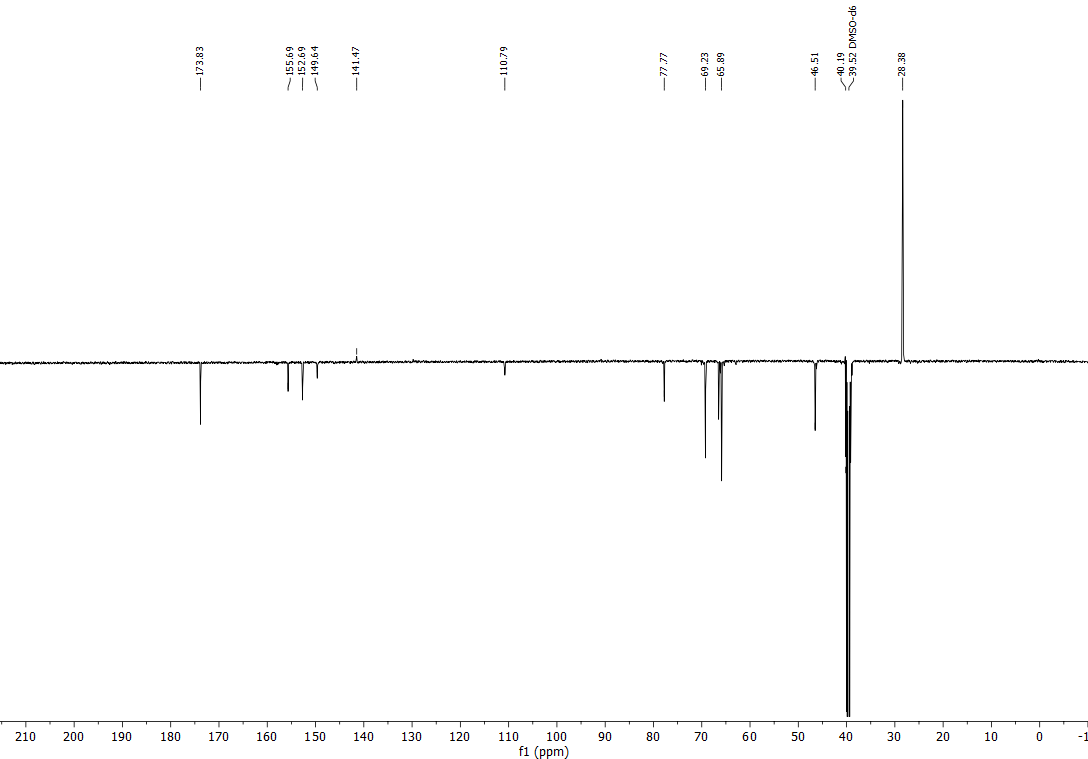


^13^C{^1^H} NMR (126 MHz, DMSO-*d*_6_, APT) of **D2**.


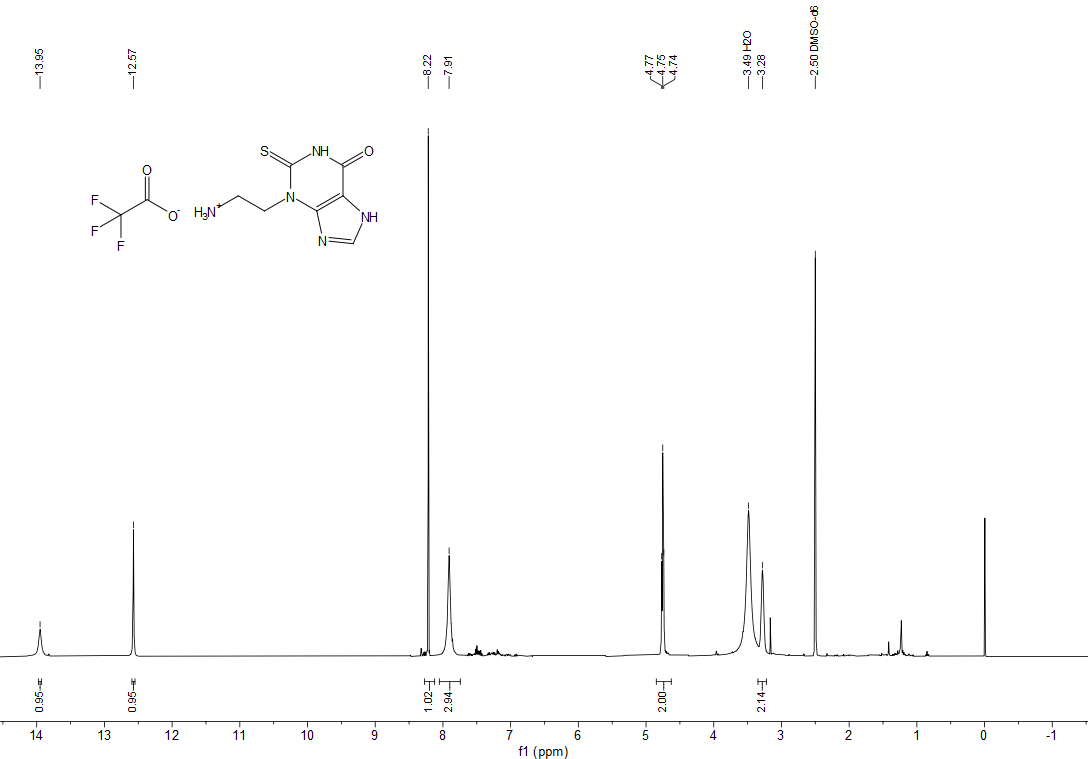


^1^H NMR (400 MHz, DMSO-*d*_6_) of **E1**.

**
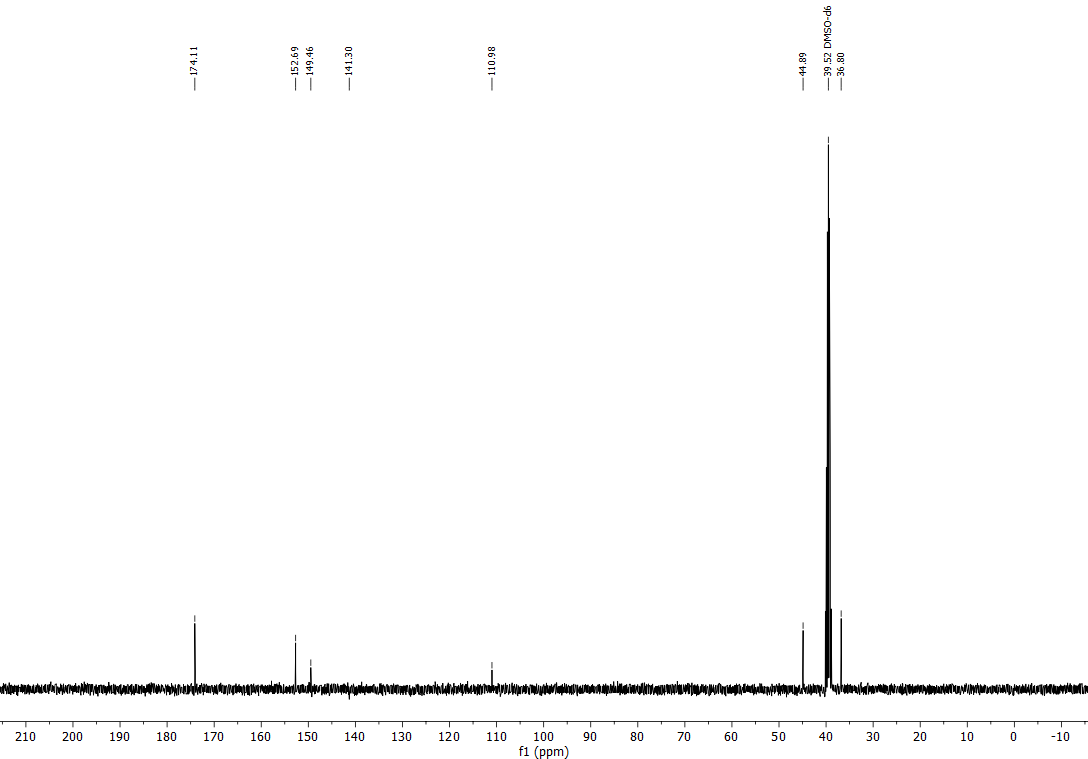
**

^13^C{^1^H} NMR (101 MHz, DMSO-*d*_6_, CPD) of **E1**.


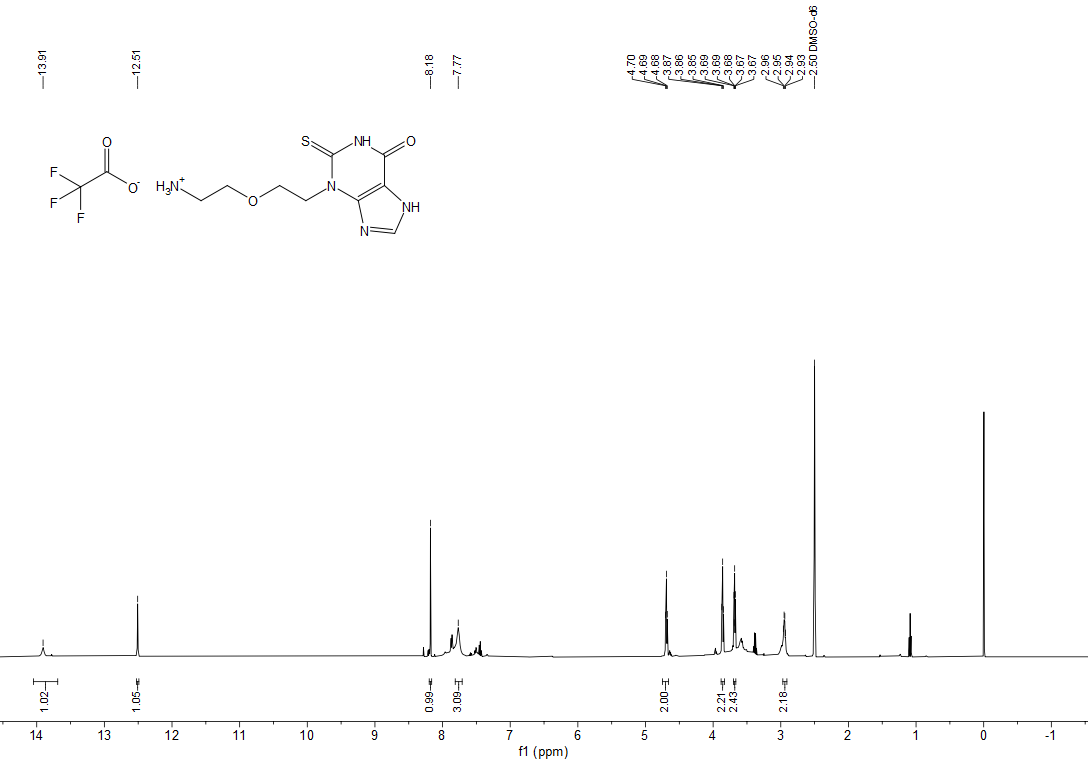


^1^H NMR (500 MHz, DMSO-*d*_6_) of **E2**.


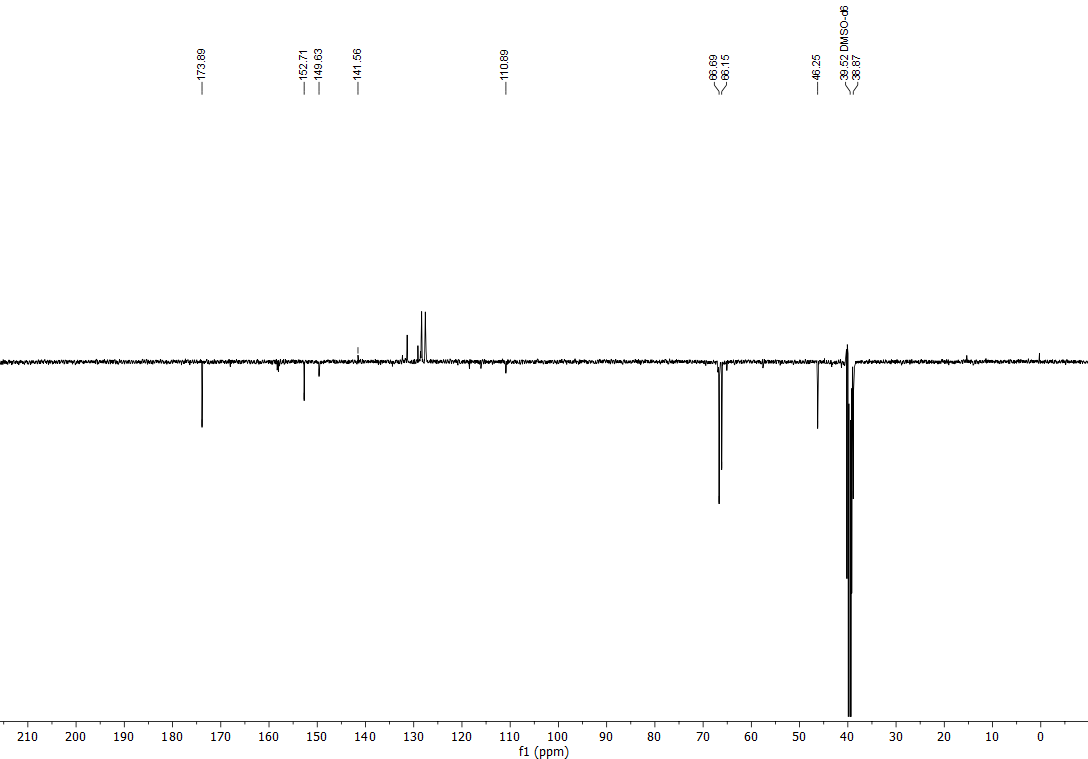


^13^C{^1^H} NMR (126 MHz, DMSO-*d*_6_, APT) of **E2**.


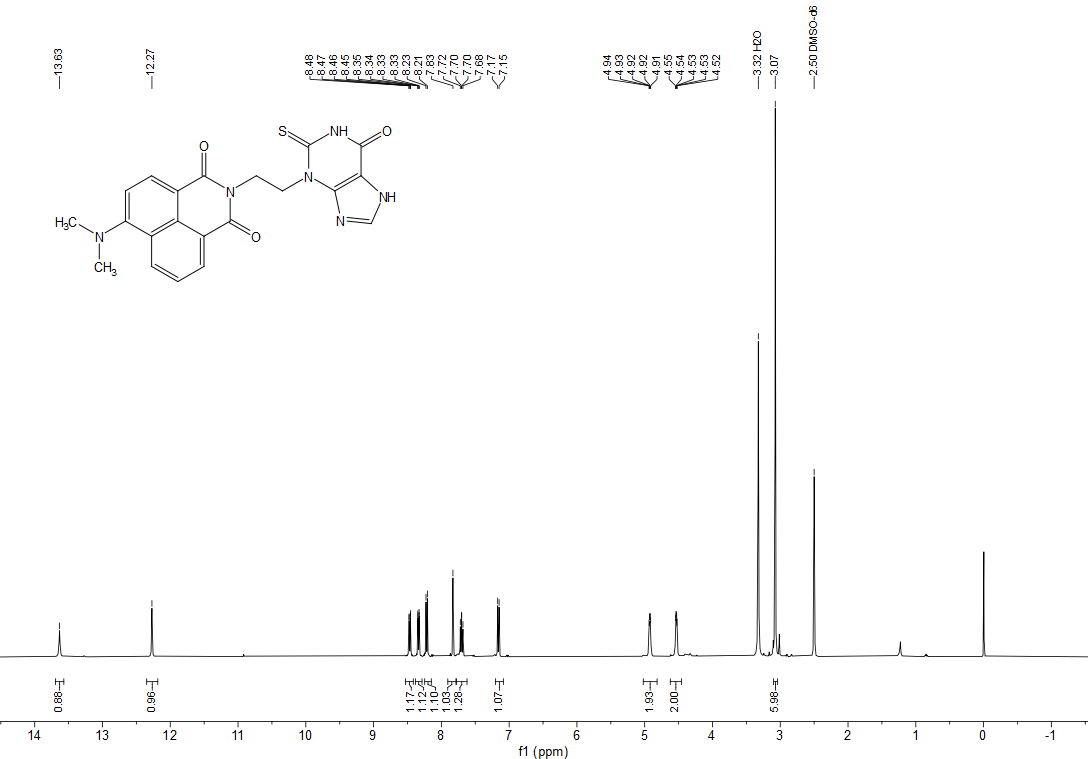


^1^H NMR (400 MHz, DMSO-*d*_6_) of probe **1**.


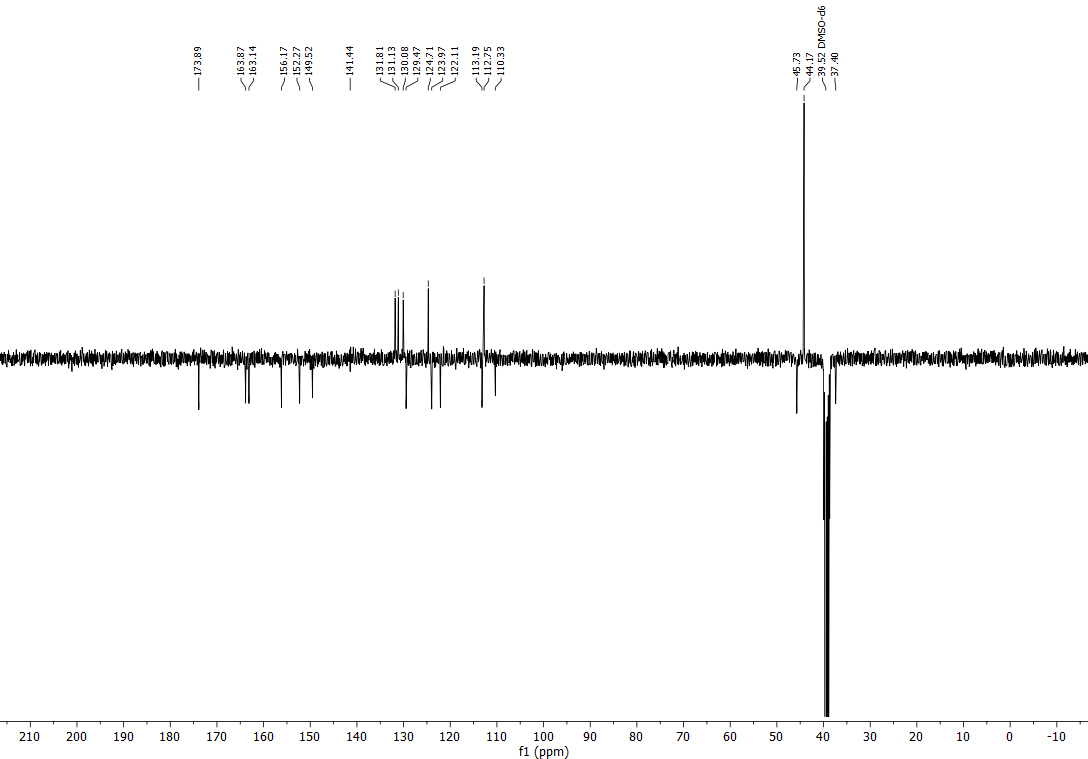


^13^C{^1^H} NMR (101 MHz, DMSO-*d*_6_, APT) of probe **1**.


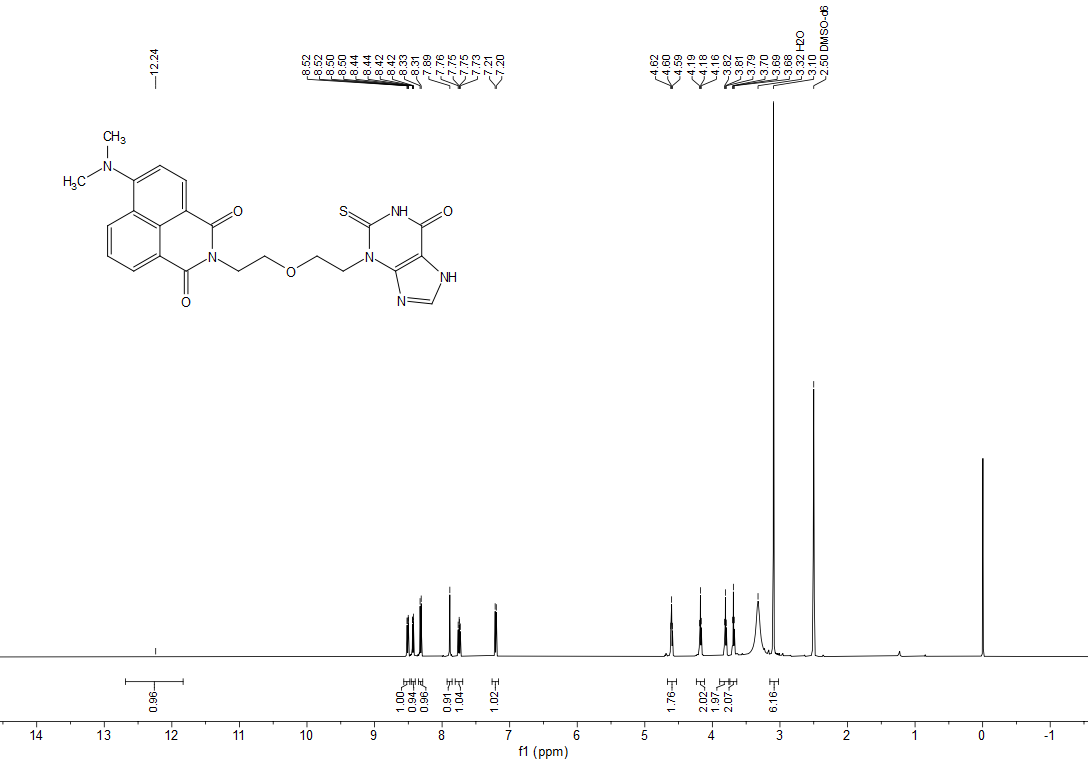


^1^H NMR (500 MHz, DMSO-*d*_6_) of probe **2**.


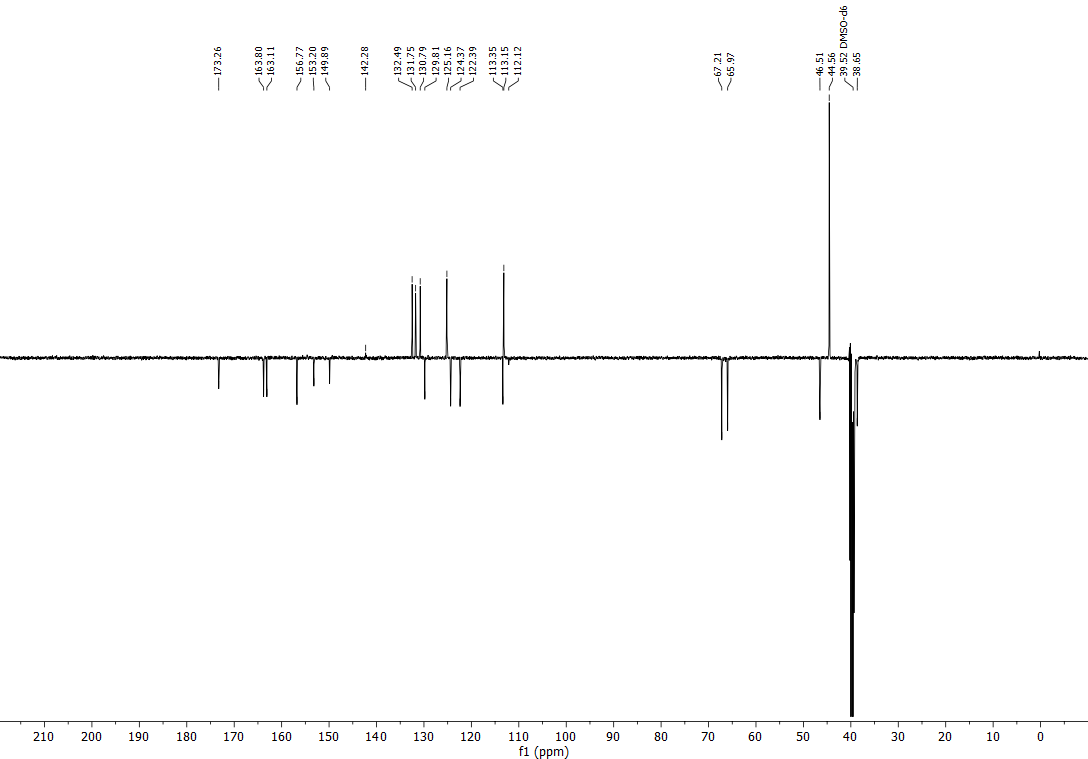


^13^C{^1^H} NMR (126 MHz, DMSO-*d*_6_, APT) of probe **2**.


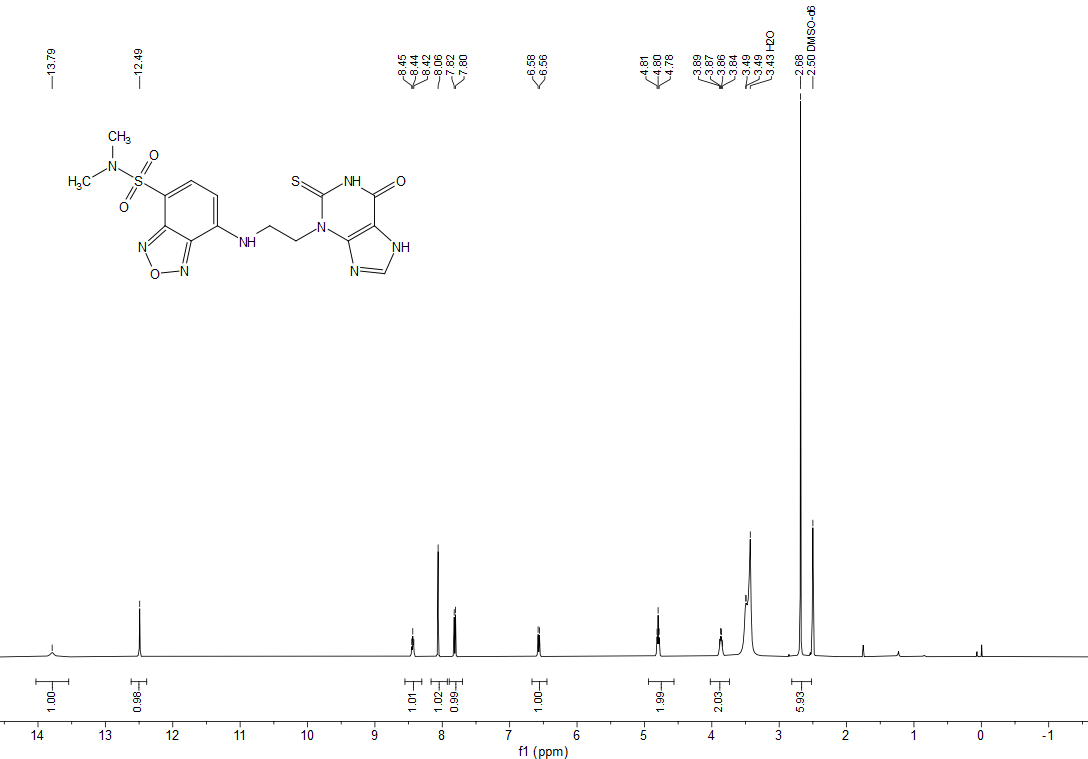


^1^H NMR (400 MHz, DMSO-*d*_6_) of probe **3**.

**
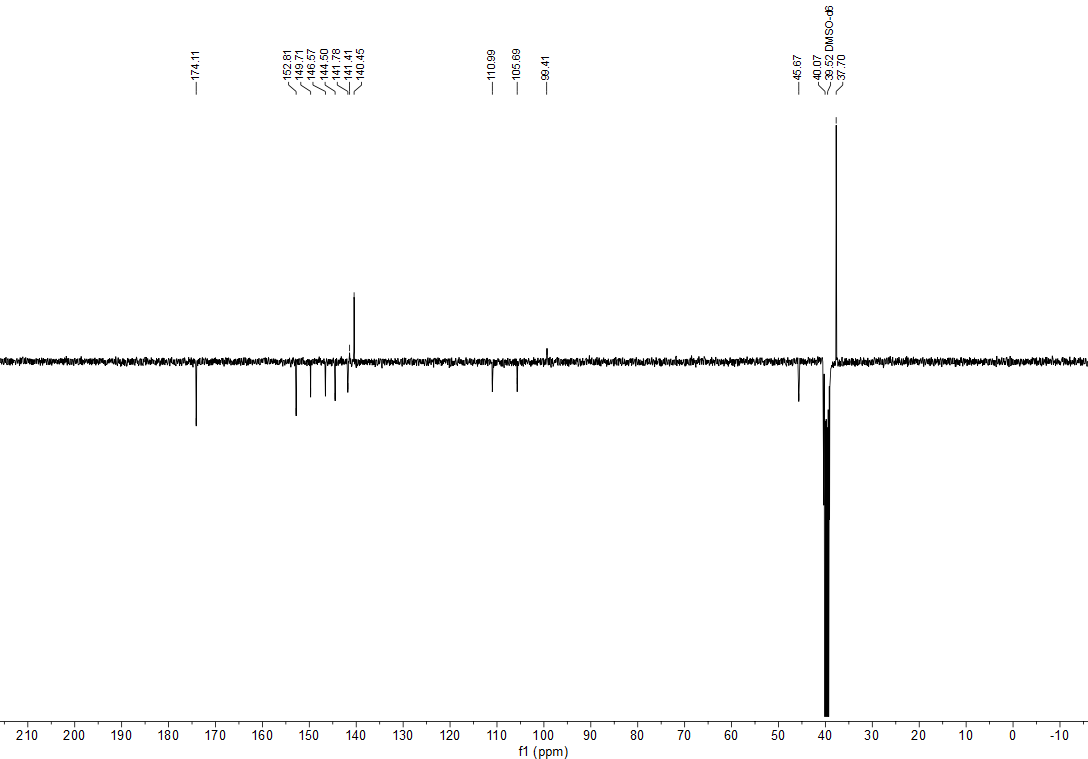
**

^13^C{^1^H} NMR (101 MHz, DMSO-*d*_6_, APT) of probe **3**.

^
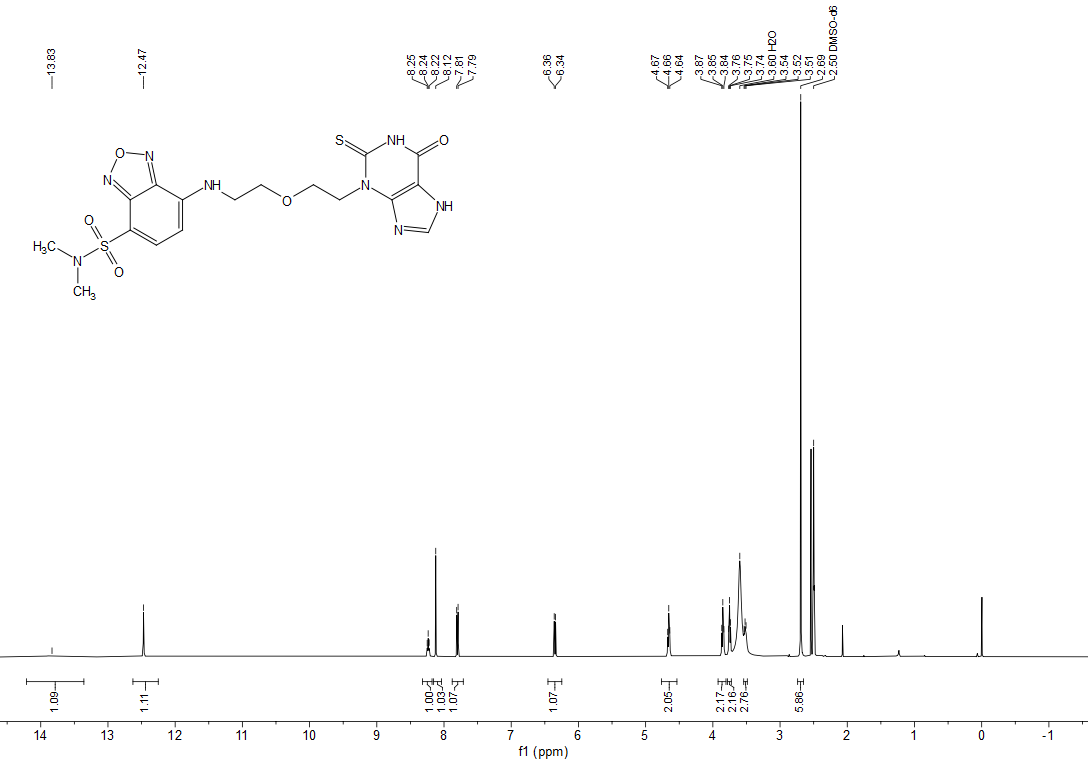
^

^1^H NMR (400 MHz, DMSO-*d*_6_) of probe **4**.

**
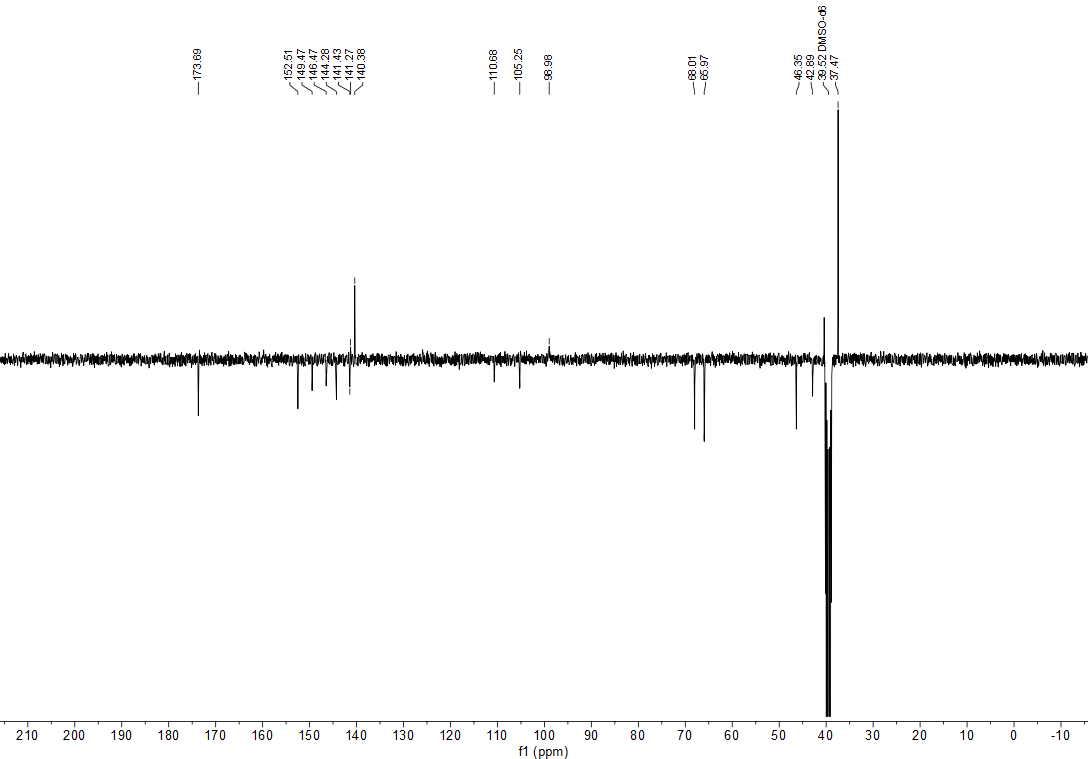
**

^13^C{^1^H} NMR (101 MHz, DMSO-*d*_6_, APT) of probe **4**.


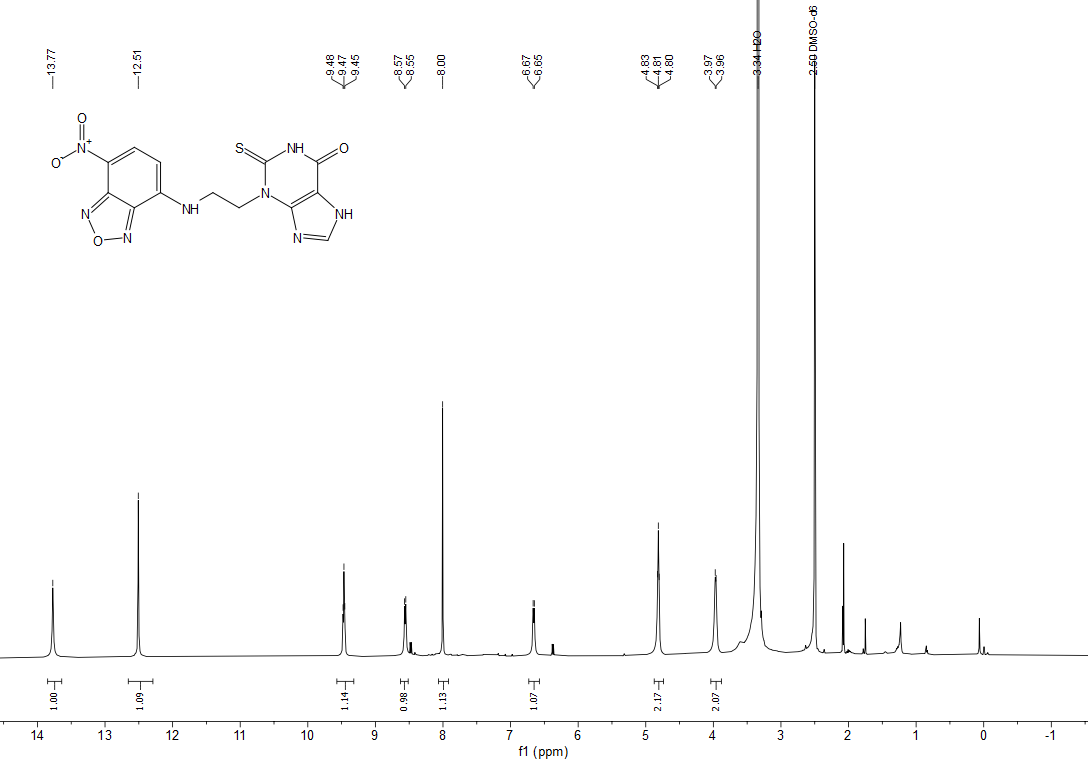


^1^H NMR (500 MHz, DMSO-*d*_6_) of probe **5**.


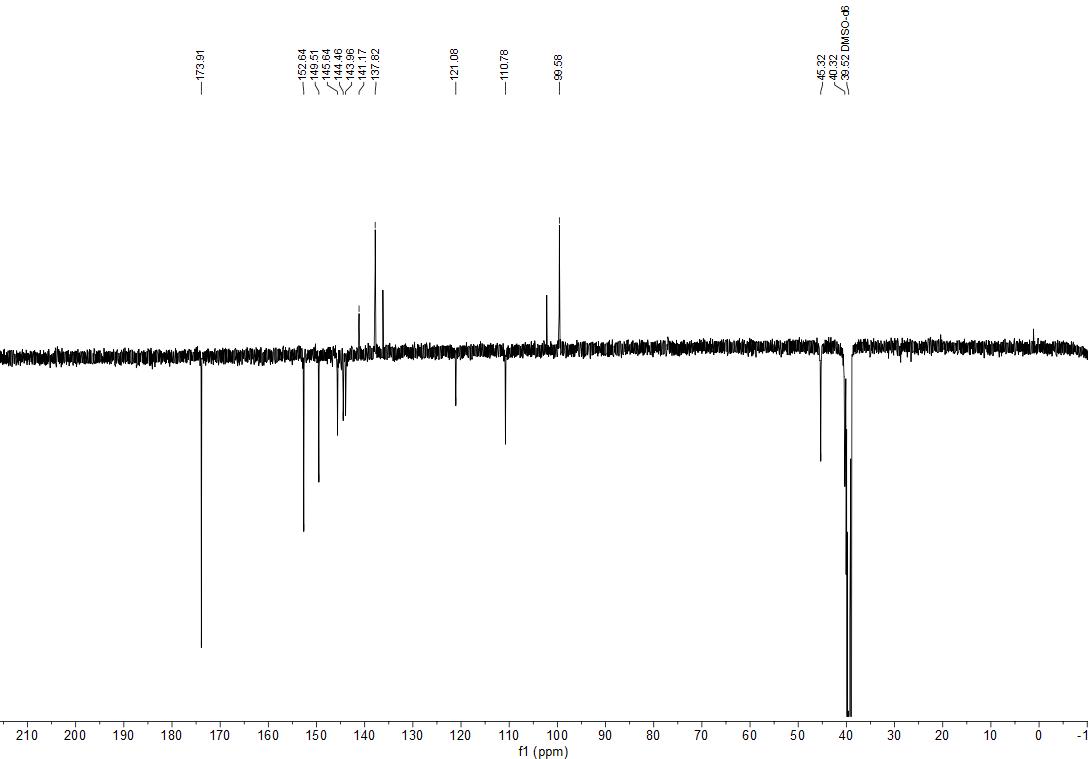


^13^C{^1^H} NMR (126 MHz, DMSO-*d*_6_, APT) of probe **5**.


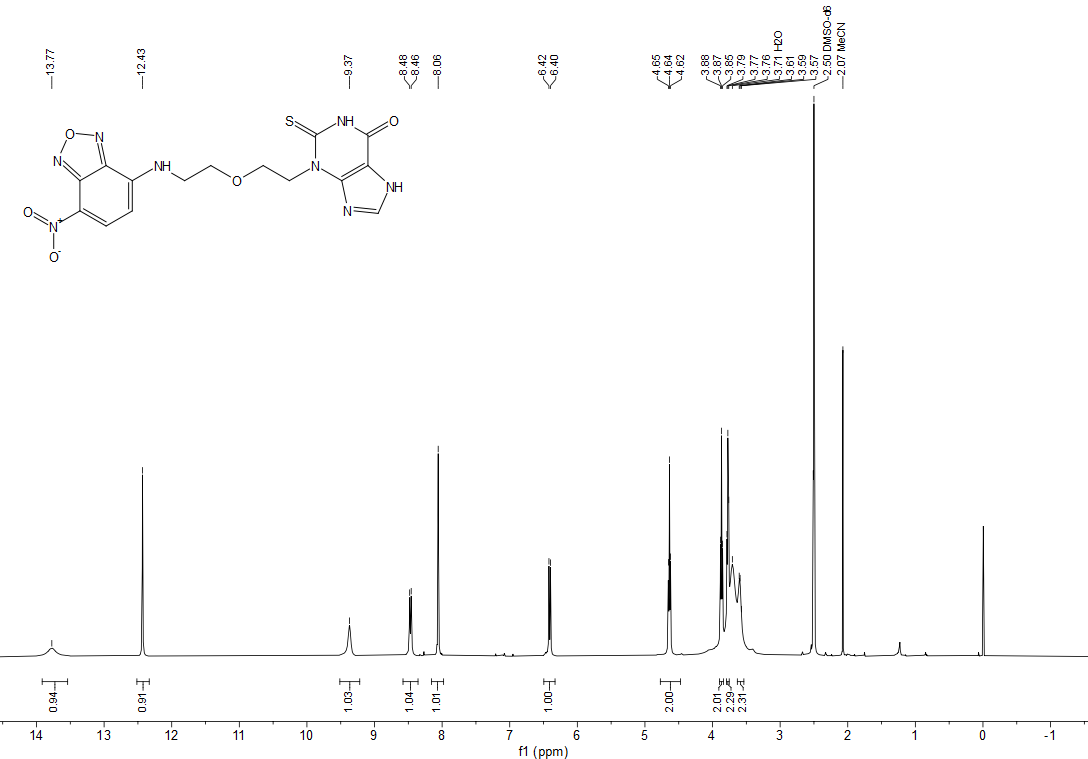


^1^H NMR (400 MHz, DMSO-*d*_6_) of probe **6**.


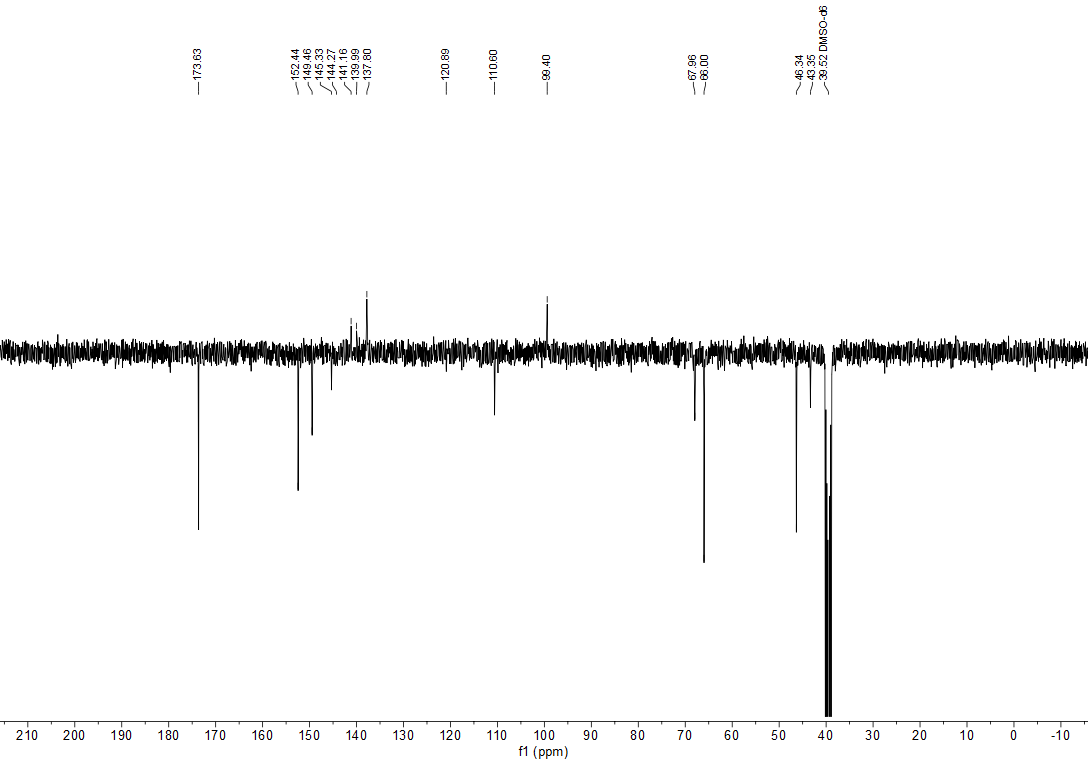


^13^C{^1^H} NMR (101 MHz, DMSO-*d*_6_, APT) of probe **6**.
