# Supplementary material for: Environment-sensitive turn-on fluorescent probe enables live cell imaging of myeloperoxidase activity during NETosis
Source: Commun Chem. 2024 Nov 12;7:262. doi: 10.1038/s42004-024-01338-5 (PMC11557929; doi:10.1038/s42004-024-01338-5)
Supplement: Supplementary file 5 — Supplementary Data 2 [file 42004_2024_1338_MOESM5_ESM.docx]

# 1. Supplementary Data 2.

## 1.1 HPLC traces.

**
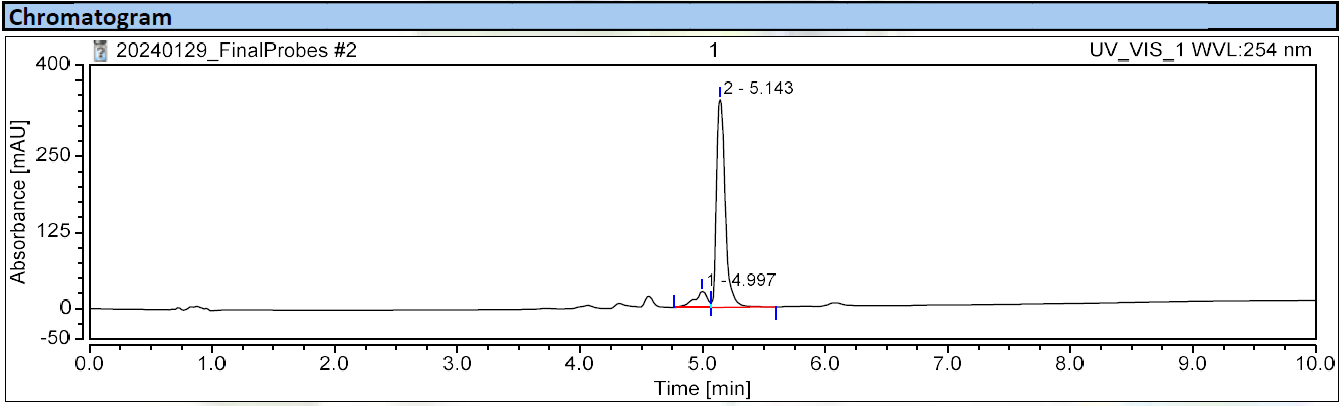
**

**
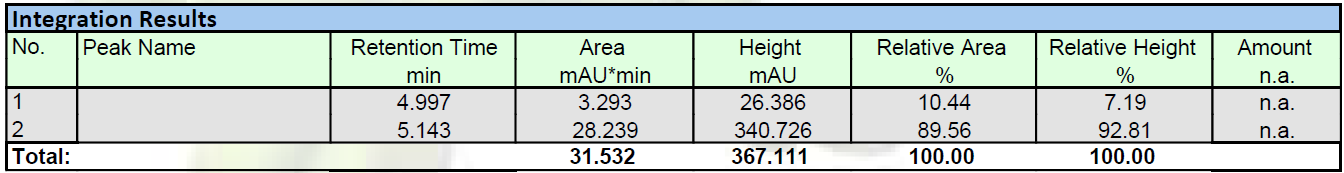
**

HPLC trace of probe **1**.

**
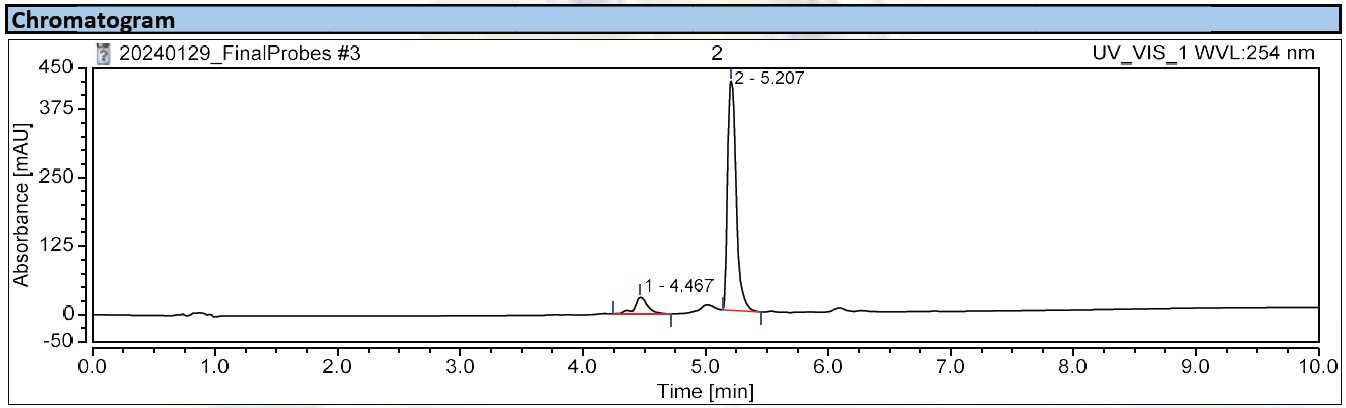
**

**
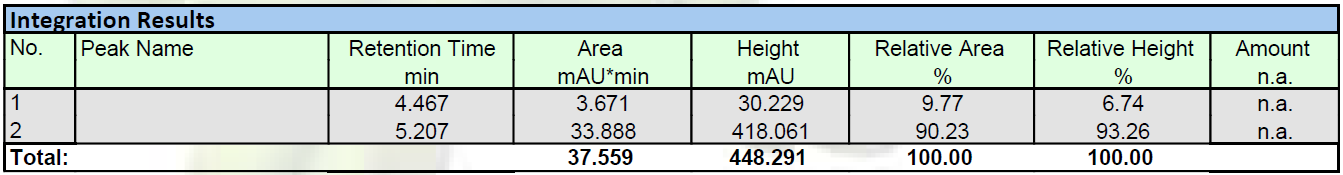
**

HPLC trace of probe **2**.

**
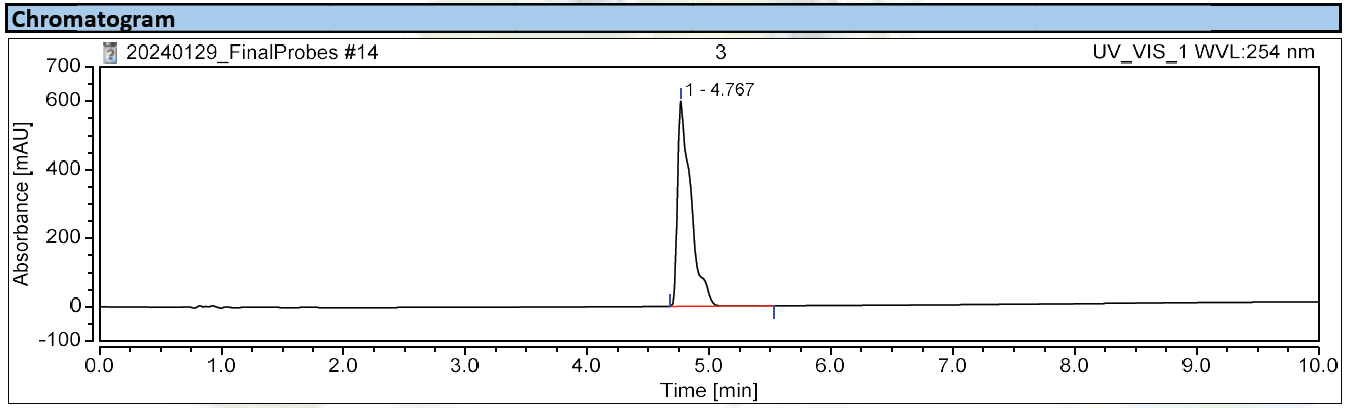
**

**
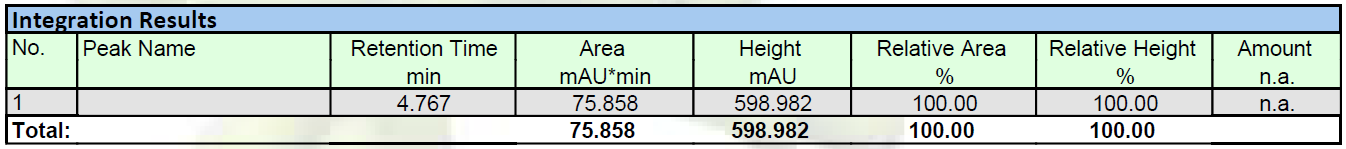
**

HPLC trace of probe **3**.

**
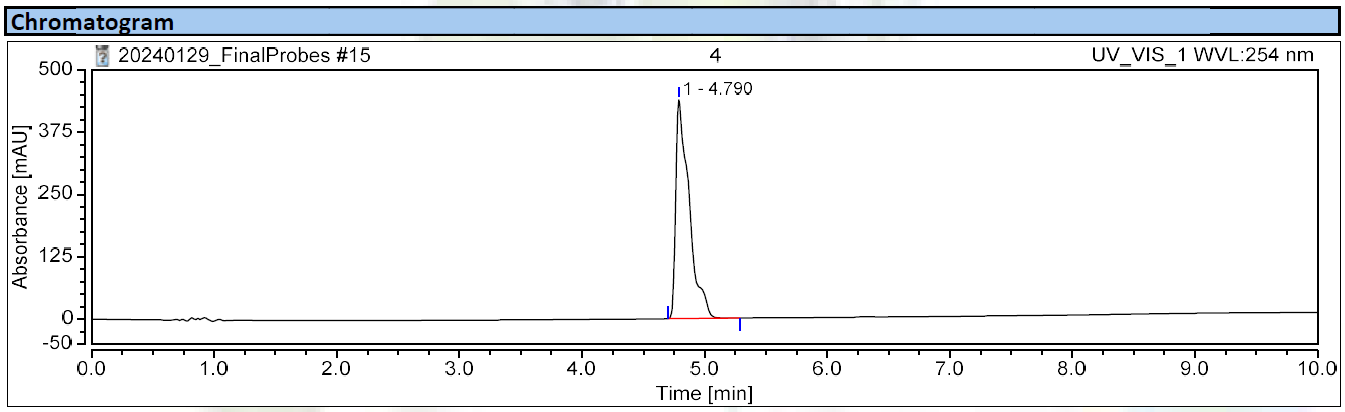
**

**
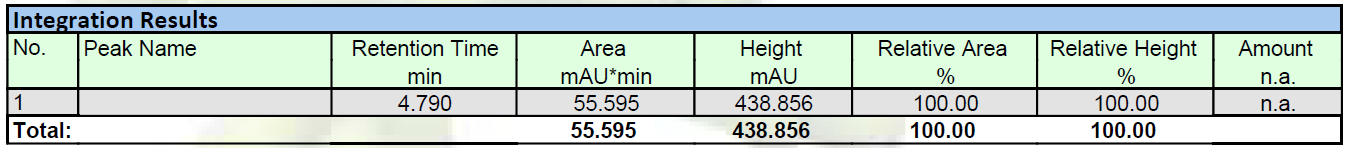
**

HPLC trace of probe **4**.

**
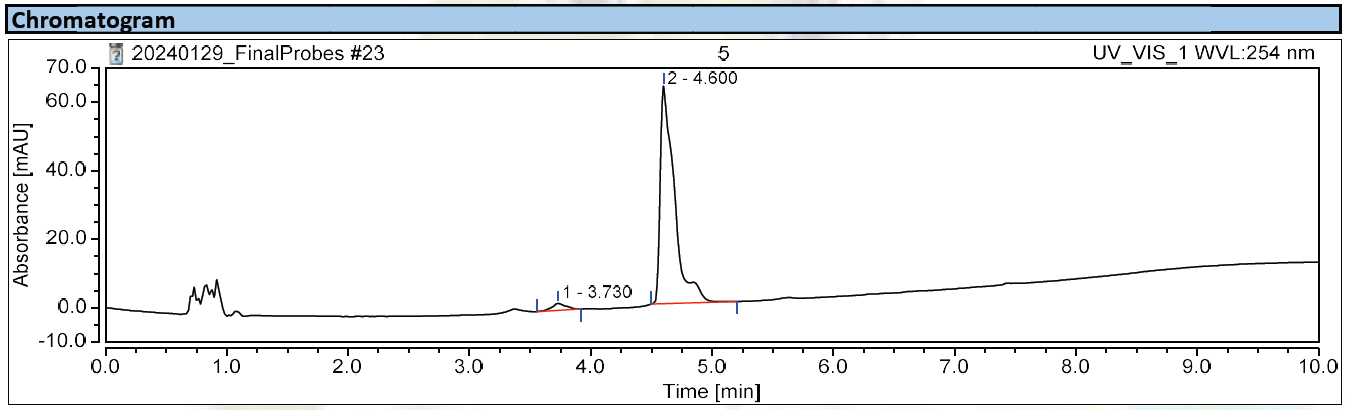
**

**
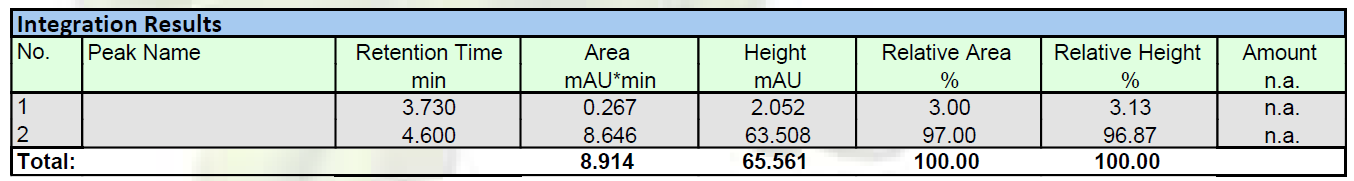
**

HPLC trace of probe **5**.

**
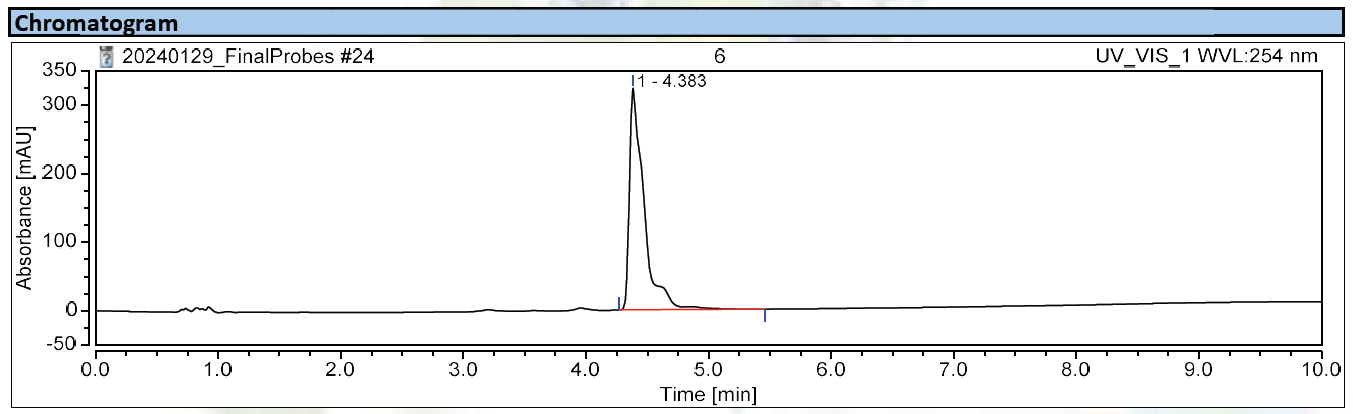
**

**
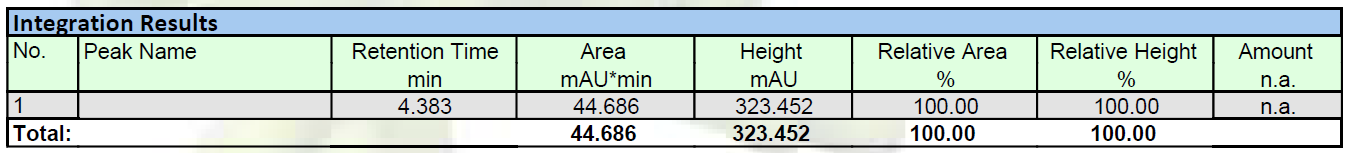
**

HPLC trace of probe **6**.
